# Concentration-Dependent Recruitment of Mammalian Odorant Receptors

**DOI:** 10.1523/ENEURO.0103-19.2019

**Published:** 2020-04-15

**Authors:** Xiaoyang Serene Hu, Kentaro Ikegami, Aashutosh Vihani, Kevin W. Zhu, Marcelo Zapata, Claire A. de March, Matthew Do, Natasha Vaidya, Gary Kucera, Cheryl Bock, Yue Jiang, Masafumi Yohda, Hiroaki Matsunami

**Affiliations:** 1 Department of Molecular Genetics and Microbiology, Duke University Medical Center, Durham, NC 27710; 2 Tokyo University of Agriculture and Technology, Tokyo 183-8538, Japan; 3 Department of Neurobiology, Duke Institute for Brain Sciences, Duke University, Durham, NC 27710; 4 North Carolina School of Science and Mathematics, Durham, NC 27705; 5 DCI Rodent Cancer Models Shared Resource, Duke University Medical Center, Durham, NC 27710

**Keywords:** chemical senses, GPCR, odor, olfactory, smell

## Abstract

A fundamental challenge in studying principles of organization used by the olfactory system to encode odor concentration information has been identifying comprehensive sets of activated odorant receptors (ORs) across a broad concentration range inside freely behaving animals. In mammals, this has recently become feasible with high-throughput sequencing-based methods that identify populations of activated ORs *in vivo*. In this study, we characterized the mouse OR repertoires activated by the two odorants, acetophenone (ACT) and 2,5-dihydro-2,4,5-trimethylthiazoline (TMT), from 0.01% to 100% (v/v) as starting concentrations using phosphorylated ribosomal protein S6 capture followed by RNA-Seq. We found Olfr923 to be one of the most sensitive ORs that is enriched by ACT. Using a mouse line that genetically labels Olfr923-positive axons, we provided evidence that ACT activates the Olfr923 glomeruli in the olfactory bulb. Through molecular dynamics stimulations, we identified amino acid residues in the Olfr923 binding cavity that facilitate ACT binding. This study sheds light on the active process by which unique OR repertoires may collectively facilitate the discrimination of odorant concentrations.

## Significance Statement

The ability of animals to discriminate odors over a range of odor concentrations while recognizing concentration-invariant odor identity presents an encoding challenge for the olfactory system. To further our understanding on how animals sense odors at different concentrations, it is important to describe how odor concentration information is represented at the receptor level. Here, we establish a sensitive *in vivo* approach to screen populations of odorant receptors (ORs) enriched in the odor-activated sensory neurons in mice. We identified comprehensive lists of enriched ORs against a 10,000-fold concentration range for two odorants. Describing the concentration-dependent activation for unique populations of ORs is fundamental for future studies in determining how individual ORs contribute to olfactory sensitivity and odor intensity coding.

## Introduction

In nature, odors coming from foods, mates and predators are dynamic in concentration. The ability to discriminate odorants at different concentrations while recognizing the same odorant across different concentrations serves nutritional, reproductive and protective purposes, allowing animals to make appropriate behavioral decisions to maximize their fitness.

The mammalian olfactory system, capable of detecting and discriminating many odorous volatile molecules, begins at the nasal passage, where millions of olfactory sensory neurons (OSNs) line the main olfactory epithelium. Odor detection is initiated by the activation of a specific set within the large repertoire of G-protein-coupled odorant receptors (ORs), encoded by ∼400 and ∼1100 intact OR genes in humans and mice, respectively (Buck and Axel, 1991; Healy et al., 1997; Zhang and Firestein, 2002; Godfrey et al., 2004; Malnic et al., 2004; Ibarra-Soria et al., 2014; Niimura et al., 2014). Mature OSNs express only one type of the available ORs at a high level (Chess et al., 1994; Malnic et al., 1999; Hanchate et al., 2015; Saraiva et al., 2015; Tan et al., 2015; Scholz et al., 2016). As OSNs expressing the same OR send convergent axonal projections to form glomeruli in the olfactory bulb, OR activation is translated into glomerular activation patterns which are further processed within the olfactory bulb and the olfactory cortical areas (Ressler et al., 1994; Vassar et al., 1994; Mombaerts et al., 1996; Rubin and Katz, 1999; Wachowiak and Cohen, 2001; Oka et al., 2006; Miyamichi et al., 2011; Sosulski et al., 2011; Storace and Cohen, 2017; Wilson et al., 2017). Odorants are represented by combinatorial codes whereby a given odorant, at a specific concentration activates a specific combination of ORs, which in turn activate a specific combination of glomeruli (Malnic et al., 1999; Rubin and Katz, 1999; Bozza et al., 2002; Oka et al., 2006; Saito et al., 2009). Previous studies found an increase in the recruitment of active ORs (in the OSNs) and glomeruli (in the olfactory bulb) in response to higher odorant concentrations (Rubin and Katz, 1999; Fried et al., 2002; Khan et al., 2010; Jiang et al., 2015; Wilson et al., 2017). There is, however, insufficient research determining the identity of these ORs and their collective responses at different odorant concentrations *in vivo*. This lack of research limited our ability to draw conclusions on how odor identity and intensity information is encoded by specific ORs.

**Figure 1.**
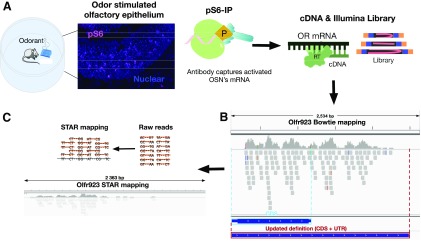
An illustration showing the updated pS6-IP-Seq method. ***A***, Following odorant stimulation, a monoclonal pS6 antibody captures mRNA from activated OSNs that is processed into an Illumina library. ***B***, Olfr923’s OR gene annotation include CDS + transcribed UTRs. ***C***, STAR aligns and quantifies OIlfr923 transcript reads at the mouse olfactory epithelium.

To address this limitation, we identified ORs activated by acetophenone (ACT) and 2,5-dihydro-trimethylthiazoline (TMT) at varying odorant concentrations *in vivo*. This was made possible through a recently developed *in vivo*-based method which identifies ORs expressed in activated OSNs via mRNA profiling (Jiang et al., 2015). This method makes use of the finding that the phosphorylation of the ribosomal protein S6 is an indicator of neuronal activation (Knight et al., 2012; Biever et al., 2015). Immunoprecipitation of ribosome-mRNA complex containing phospho S6 (pS6-IP also known as phosphoTRAP) followed by next-generation sequencing (pS6-IP-Seq) identifies OR mRNAs present in activated OSNs (Fig. 1).

Besides pS6-IP-Seq, there are two additional approaches to study OR responses *in vivo*: one is the Kentucky method based on activation-induced S100a5 expression (McClintock et al., 2014) and another is the DREAM technique (deorphanization of receptors based on expression alterations of mRNA levels) which is based on OR-specific transcriptional downregulation after prolonged odor stimulation (von der Weid et al., 2015). It has been previously reported that DREAM and pS6-IP-Seq identified overlapping, but not identical sets of ORs (Jiang et al., 2015; von der Weid et al., 2015).

In this study, we used a combination of *in vivo*, *in situ*, and *in silico* approaches to investigate ORs with distinct sensitivities to the tested odorants. In addition, we compared data obtained by pS6-IP, DREAM and heterologous expression methods to clarify the degree of consistency in the ACT responsive ORs identified by these methods. We examined Olfr923, which we identified to be one of the most sensitive ACT ORs based on our pS6-IP-Seq data. To this end, we created a gene knock-in mouse line to test activation of the Olfr923 glomeruli in the olfactory bulb. Additionally, we performed molecular modeling to evaluate ligand binding and amino acid residues involved in ACT’s activation of Olfr923.

## Materials and Methods

### Animals

All animal procedures were performed in accordance with the Duke University animal care committee’s regulations. All experiments in this paper were conducted on both male and female mice. C57BL/6J and B6.Cg-Gt(ROSA)26Sor tm9(CAG-tdTomato)Hze/J were obtained from the Jackson Laboratory and crossed in the mouse facility. C57BL/6J mice of 20–22 d of age were used for pS6-IP-Seq, RNA-Seq and staining experiments. Littermates of the same sex were used for each pS6-IP-Seq replicate (*n* = 3) and data from both sexes were pooled to avoid sex-specific genes being enriched in a given odor exposure. Although we did not formally test between the two sexes for differences, we did not observe any obvious differences between males and females in the experiments.

### Odor exposure

Odorant exposures of ACT or TMT were performed on C57BL/6J mice as described (Jiang et al., 2015). Mice were habituated in a disposable paper container for 1 h and exposed to an odor cassette for 1 h. The starting concentrations of odorants used for stimulations contained 10 μl of tested odorant diluted with dW (v/v). Diluted odorants were spotted individually onto a 2 × 2-cm blotting paper and placed into a Uni-cassette (Sakura) for odor stimulation. The mice stimulation environment was inside a fresh paper container with lid (International Paper) within the fume hood.

### pS6-IP-Seq

Following odor stimulation, the mouse was sacrificed, and the dissected olfactory epithelium was processed for RNA library in reference to the method described by Jiang et al. (2015), with some modifications. Pieces of freshly dissected olfactory epithelium were homogenized and centrifuged. 6 μl of a monoclonal phospho-S6 ribosomal protein (Ser240/244; D68F8) XP rabbit mAb (Cell Signaling Technologies) was added to the supernatant, and incubated with rotation for 1.5 h at 4°C. Protein A Dynabeads (Invitrogen) were added to the antibody mixture and incubated for another hour with rotation at 4°C. After incubation and washing, the bead-captured RNA was eluted and purified with RNeasy Micro kit (QIAGEN). 10 ng of starting RNA was used to make cDNA libraries with the SMART-Seq v4 kit (Clontech) with 11 cycles of PCR. cDNA was purified with AMPure XP beads (Beckman Coulter) and 0.5 ng was used to make cDNA libraries using the Nextera XT DNA Library Preparation kit (Illumina). 50 base sequencing was performed on the HiSeq 2500 System (Illumina), obtaining 15–38 million reads per sample. Sequence reads were mapped using STAR alignment (Dobin et al., 2013) against the entire transcriptome and newly annotated ORs [untranslated regions (UTRs) annotations included for all genes]. Reads mapped onto 1088 intact OR genes (297 ORs with pseudogene labels removed) were used for the purpose identifying odorant activated OSNs based on differential expression analysis with edgeR (Anders and Huber, 2010; Robinson et al., 2010; McCarthy et al., 2012; for R code, see Extended Data 1). ORs with false discovery rate (FDR) corrected *p* value <0.05 and a positive fold change were considered significantly enriched. Raw reads and quantification results can be accessed at GEO: data submitted and processing.

10.1523/ENEURO.0103-19.2019.ed1 Extended Data 1 R code for differential expression analysis. Download Extended Data 1, ZIP file.

### Code accessibility

R code for differential expression can be accessed via GitHub: https://github.com/serenehu/eNeuro.pS6IP.

### OR mapping and annotation

Based on the Jiang et al., publication, mRNAs captured by pS6-IP were mapped based on the *Mus musculus* (house mouse) genome assembly GRCm38 (mm10) coding exons using Bowtie (Jiang et al., 2015). OR transcript definitions were re-annotated to include 5′ and 3′ UTRs with the coding sequence. This is based on Ibarra-Soria et al’s work which included new annotations for 1248 mouse ORs (Ibarra-Soria et al., 2014). University of California Santa Cruz genome assembly mRNA sequences were used to visualize transcripts that mapped onto coding and non-coding parts of the exons for all genes (Kent et al., 2002). 58 ORs listed as coding exons in Ibarra-Soria’s definition were replaced in the new UTR included OR definitions. Olfr151 was misannotated and corrected as Olfr160; four ORs which were not yet annotated were added from the refGene database (O'Leary et al., 2016). In total, we included the gene definitions of 1385 ORs (pseudogenes included) in our new pS6-IP-Seq pipeline. To analyze the Illumina sequencing read files, we created a customized GTF annotation file. BLAT v. 35 (Kent, 2002) which was used to align intact OR sequences to the mouse genome represented by the Mus_musculus.GRCm38.dna_sm.primary_assembly.fa.gz file from Ensembl release 96 (April 2019). A custom R script was used to remove hits that did not overlap with the coding region of the queried OR and format the results in GTF format. OR pseudogenes and non-OR genes were defined using the ensembl standard file Mus_musculus.GRCm38.96.gtf.gz file. Snakemake v3.5.5 (Köster and Rahmann, 2012) was used to process read files through alignment and quantification. STAR v2.7.0d (Dobin et al., 2013) was used to generate a genome index using the Ensembl Mus musculus primary assembly genome sequence and custom GTF annotation file. Reads were aligned to this genome index using STAR with default options except for –quantMode TranscriptomeSAM which maps genome alignments to transcript coordinates. STAR output transcriptome SAM files were quantified using RSEM v1.3.1 (Li and Dewey, 2011) using default options to generate gene and transcript level counts for differential expression.

### RNA-Seq from dorsal and ventral olfactory epithelium

After the dorsal and ventral olfactory epithelium were dissected, RNA was extracted using the TRIzol reagent (Invitrogen) followed by clean up with RNeasy Mini (QIAGEN). A total of 1000 ng of RNA were used for cDNA libraries using the SMART-Seq v4 kit (Clontech), with two cycles of PCR. cDNA was purified with AMPure XP beads (Beckman Coulter) and 0.5 ng was used to make cDNA libraries using the Nextera XT DNA Library Preparation kit (Illumina). The sequenced reads were then mapped and analyzed using the same bioinformatics pipeline as described above for pS6-IP-Seq. Out of the 117 unclassified ORs, 45 ORs were unclassified due to low expression. The vast majority of the remaining ORs (70/73) are assigned to zonal index positions between 1.5 and 2.5 (1 being most dorsal and 5 being most ventral) in Tan and Xie (2018), suggesting that the these unclassified ORs are expressed in areas close to the dorsal/ventral boundary (Extended Data Fig. 2-1).

**Figure 2.**
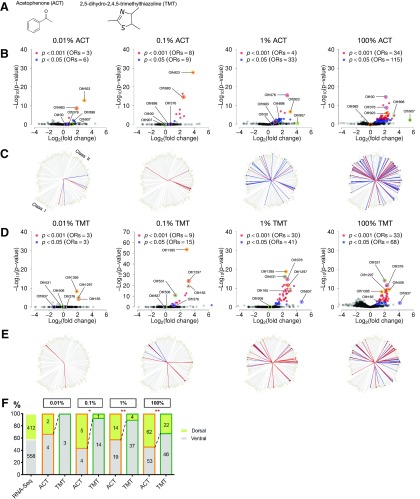
OR repertoires expand with increasing ACT and TMT concentrations. ***A***, Chemical structure of ACT and TMT. ***B***, ***D***, Volcano plots showing the log_2_ fold change and -log_10_ *p* value (FDR corrected) of OR genes from 0.01% to 100% starting concentrations of ACT and TMT. OR differential expression values are based on pS6-IP-Seq comparing odorant stimulated and unstimulated C57BL6 mice. ORs significantly enriched by pS6-IP-Seq with positive fold change are colored in blue and red based their *p* values (FDR corrected). *N* = 3 mice were used for each odorant condition. ***B***, Six ACT responsive ORs: Olfr923, Olfr983, Olfr376, Olfr898, Olfr907, and Olfr30 are labeled. ***D***, Seven TMT responsive ORs: Olfr1395, Olfr1297, Olfr165, Olfr531, Olfr506, Olfr837, an Olfr376 are labeled. ***C***, ***E***, Protein sequence distance trees generated using similarity based on pairwise distances from aligned OR protein sequences. Each end of the branches represents an OR placed alongside its closest neighbors. Both class 1 (bottom left ¼ the tree) and class 2 (remaining ¾ of the tree) ORs are shown. ORs significantly enriched by ACT and TMT at each odorant concentrations are labeled in red (FDR corrected *p* < 0.001) and blue circles (FDR corrected *p* < 0.05). ***F***, Stacked bar graphs showing the zonal location of ACT and TMT activated ORs across various concentrations. Orange and green colors outline ACT and TMT graphs, respectively; *p < 0.05, **p < 0.01, by Fisher’s exact test (ACT compared with TMT). *N* = 3 mice olfactory epithelium were used in RNA-Seq to determine OR zonal positions. See Extended Data Figure 2-1 for zonal index comparisons. See Extended Data Figures 2-2, 2-3, 2-4, 2-5, 2-6, 2-7, 2-8, 2-9, 2-10, 2-11, and 2-12 for differential expression data supporting this figure.

10.1523/ENEURO.0103-19.2019.f2-1 Extended Data Figure 2-1 *A*, OR dorsal and ventral zonal distribution data based on RNA-Seq is correlated with the zonal index data by Tan et al. (2015) where their zonal index increases progressively from dorsal to ventral; ***p < 0.001 by Mann–Whitney test (dorsal compared to ventral). ***B***, The OR zonal index distribution for ORs activated by ACT or TMT shows statistical difference between odorants; **p < 0.01, ***p < 0.001 by Fisher’s exact test (ACT compared to TMT). Download Figure 2-1, EPS file.

### *In situ* hybridization and immunohistochemical staining

Fluorescent *in situ* hybridization (FISH) and immunohistochemical staining were performed in reference to Jiang et al. (2015) with some modifications. Briefly, 18-μm frozen sections of the mouse snout containing the olfactory epithelium were fixed in paraformaldehyde and pretreated. Digoxigenin (DIG)-labeled complementary RNA probes were hybridized to the tissue sections overnight at 58°C. Unless specified, we used open reading frame of ORs for the RNA probes. We designed a 3’UTR specific probe for the labeling of Olfr376 because of open reading frame sequence similarity to other ORs, and the primers used for cloning into pCI vector were as follows: AAACGCGTCAGTAATATTTTAA CACTGA (forward) and AAGCGGCCGCCTGAGTGTACAGTTTTTGAG (reverse). Following washes, the sections were incubated for 45 min with a horseradish peroxidase (HRP)-conjugated antibody (Roche, 1:1000 in blocking solution) against DIG. Hybridization signals were labeled with a 10-min incubation of tyramide signal amplification (TSA) solution. For immunostaining, the FISH-labeled sections were incubated with a polyclonal rabbit anti-phospho-S6 (244/247; Thermo Fisher Scientific, 1:300 dilution in blocking solution) overnight at 4°C. Following washes, a 45-min incubation with the donkey Cy3-conjugated anti-rabbit IgG (Jackson ImmunoResearch Laboratories, Inc., 1:200) was used to visualize pS6 proteins. Olfactory epithelium images were taken using the Zeiss Axiocam MRm and upright inverted fluorescent microscope with ApoTome functionality at 200× magnification. The filter sets used were as follows: Zeiss filter set #38 for fluorescein, #43 for Cy3, and #49 for bisbenzimide. ImageJ was used in the quantification of colocalization signals, where pS6 pixel intensity of DIG-positive OSNs was subtracted from the average pixel pS6 intensity of no odor exposed OSNs labeled with the same OR probe. The resulting normalized intensities were quantified and compared using one-way ANOVA with Dunnett’s test.

### Olfr923-CRE transgenic mouse

#### Targeting of the *Olfr923* locus

A targeting vector expressing Cre under control of the Olfr923 locus was generated by BAC recombineering (Liu et al., 2003) in SW102 cells (Warming et al., 2005). Briefly, an IRES-Cre-frt-neomycin-frt cassette was introduced by homologous recombination into BAC clone bMQ186d17 (SystemBioSciences) containing 147 kb of mouse chromosome 9 immediately downstream of the TGA stop codon within the Olfr923 coding region. Next, the targeting vector containing the IRES-Cre-Neo flanked by homology arms was retrieved by gap repair from the BAC into plasmid pL253. The resulting targeting vector was linearized with NotI restriction endonuclease and purified by phenol:chloroform extraction and ethanol precipitation. Murine ES cell line was electroporated with a linearized targeting vector containing 7 kb of 5′ homology and 2 kb of 3′ homology to the endogenous *Olfr923* locus (G4 mES Cells were acquired from Samuel Lunenfeld Research Institute Mount Sinai Hospital).

### PCR screening of targeted ES cells

Allele-specific primers were used to screen ES cells from genomic DNA for homologous recombination across the 5′ long arm (F1: 5’-ACA GTG CAC AAA ACT AAC CCC-3’, R1: 5’-CTA CCG GTG GAT GTG GAA TGT GTG-3’) and 3′ short arm (F2: 5’-CTA TCG CCT TCT TGA CGA GTT CTT C-3’, R2: 5’-CAA CAG AAG ATG GAC TTC AGA AC-3’) by PCR.

### ES targeting

A total of 15 million G4 ES cells (Nagy et al., 1993) passage 13, were electroporated with 35 μg of targeting construct in a 4 mm gap cuvette using a Bio-Rad Genepulser II with Bio-Rad Capacitance Extender, set at 0.25 KV, 0.5 UF × 1000 for two pulses. Transfected cells were plated onto four 10 cm plates with neomycin resistant feeder cells. Targeted cells were fed selection medium with 250 μg/ml active G418 Geneticin at 24 h after transfection, followed by the addition of two Um ganciclovir to the selection medium at 48 h. Selection medium with G418/Ganc was changed once a day until clones were picked on day 7 after transfection. A total of 142 clones were picked and expanded on 96-well plates. DNA was analyzed by PCR for homologous recombination on the 3′ end. 24/142 clones were identified as positive. Eighteen clones were expanded to the six-well size plate, then cryopreserved in duplicate aliquots. Further analysis was done on genomic DNA prepared from these expanded clones by 3′ and 5′ Southern blotting and PCR to confirm homologous recombination. Confirmed clones were injected into an ICR morulae host embryo (Nagy et al., 1993). Five clones produced a high percentage of agouti coat color chimeras which were used for breeding and expansion of the colony for this study.

### Olfactory bulb immunohistochemistry

Three- to six-week-old mice were habituated for 1 h and then received either control or ACT stimulation by spotting 10 μl of freshly made 0.01% ACT onto blotting paper placed in an odor cassette for 1 h. Control conditions consisted of placing clean blotting paper into an odor cassette. The mice were sacrificed and olfactory bulbs were collected into cold fixative solution consisting of 1× PBS, 4% paraformaldehyde for 2 h with gentle shaking. Subsequently, olfactory bulbs were washed with cold 1× PBS three times and placed into cryoprotective solution consisting of 1× PBS, 30% sucrose, overnight with gentle shaking (Nutator S0500-VWR). The next day, sunken olfactory bulbs were collected and placed into optimal cutting temperature (OCT) compound and flash frozen with liquid nitrogen. Olfactory bulbs were then sectioned at 20-μm sections using a cryostat. Detection of endogenous TdTomato fluorescence using 554/581 nm (excitation/emission) light was used to confirm glomerulus presence whilst sectioning. For fluorescent labeling of c-Fos-positive periglomerular cells, olfactory bulb sections were immersed in 1× PBS for 5 min at room temperature. Slides were then blocked with 4% donkey serum (Jackson ImmunoResearch 017-000-121) and 1% Triton X-100 for 1 h at room temperature. Subsequently, slides were incubated with the primary anti c-Fos antibody (1:400, 9F6 Cell Signaling 2250) overnight at 4°C. The next day, slides were washed in 1× PBS three times at room temperature. Slides were then incubated with secondary donkey anti-rabbit Alexa Fluor 488 (1:200, Jackson Laboratories) diluted in 1× PBS, 5% skim milk for 1 h. Slides were then washed in 1× PBS two times, stained with bisbenzimide (1:1,000,000) for 5 min, washed with 1× PBS, distilled water, and mounted with mowiol. Glomeruli with the greatest density of tdTomato expressing OSN terminals were imaged and periglomeruli cells in the immediate vicinity of the Olfr923 glomerulus were counted. C-Fos signal intensities were quantified as follows. We obtained 200× magnification Z–stacked images with 2-μm intervals using the Zeiss Axiocam MRm and upright inverted fluorescent microscope. The filter sets used were as follows: Zeiss filter set #38 for Alexa Fluor 488, #43 for tdTomado, and #49 for bisbenzimide.

### GloSensor assay

The liquid-stimulation GloSensor assay was conducted as previously described (Kida et al., 2018) with slight modifications. Hana3A cells were plated onto 96-well plates and placed into a 5% CO_2_ incubator overnight; 18–24 h after plating, the cells were transfected with 80 ng/well of Olfr923-pCI plasmid, 5 ng/well of RTP1S and 10 ng/well of GloSensor plasmid (Promega) and placed into a 5% CO_2_ incubator overnight; 18–24 h later, the medium was replaced with 50 μl of HBSS (Invitrogen) containing 10 mM HEPES and 1 mM glucose (wash step). This was followed by adding 30 μl of the HBSS containing GloSensor cAMP Reagent (Promega). Plates were kept in a dark place at room temperature for 2 h to equilibrate the cells. ACT and heptanal were diluted individually in DMSO (Sigma-Aldrich) to 100 mM working stocks, and further diluted in the GloSensor buffer (Promega) for dose–response experiments; 30 μl/well of diluted liquid odorants were added and the test plate was immediately inserted in the plate reader. The luminescence in each well was measured at 90-s intervals for six cycles. All luminescence values were normalized by dividing by the value obtained from the wells transfected with the empty pCI vector in the same cycle.

### Homology modeling

The protocol follows two previously published methods with an optimal alignment which fulfills constraints provided by >100 mutants from the literature (Charlier et al., 2013; Yu et al., 2015). Four experimental non-olfactory GPCR structures (1U19, 3ODU, 2YDV, and 2LNL) were selected as templates to build Olfr923 by homology modeling using Modeller (Eswar et al., 2006). The percentage of identity for the seven transmembrane domains of Olfr923 with respect to the four templates were as follows: 22.02% with Rhodospin (1U19), 13.68% with CxCR4 (3ODU), 19.87% with A2a (2YDV), and 25.00% with CxCR1 (2LNL). The N-terminal structure was omitted to avoid perturbing the modeling protocol. Five models were obtained and we kept the one that was consistent with several additional structural constraints (no large folded structure in extra-cellular loops should be observed, all TMs and H8 folded as α-helices, a short α-helix structure between TM3 and TM4).

### Docking of ligand

ACT and heptanal structures were prepared with the antechamber module of AMBER with AM1-BCC charges. They were docked into the receptor cavity with Autodock Vina with an exhaustiveness parameter of 50. An exhaustiveness of 15 produced the same resulting poses (Trott and Olson, 2010). For each ligand, we considered simulations with the lowest binding free energy for both molecules.

### Molecular dynamics simulation

All systems were then embedded in a model membrane made up of POPC lipids solvated by TIP3P water molecules using Maestro (Schrödinger, 2013). The total system was made up of ∼77,000 atoms. For all systems in this article, molecular dynamics simulations were performed with sander and pmemd.cuda modules of AMBER with the ff03 force-field for the protein the gaff.lipid force-field for the membrane, and the gaff force-field for the ligands (Case et al., 2014). Bonds involving hydrogen atom were constrained using SHAKE algorithm. Long-range electrostatics interactions were handled with the particle mesh Ewald (PME) method. The cutoff for non-bonded interactions was set to 8 Å. Temperature was maintained constant with a Langevin thermostat with a collision frequency of 2 ps^−1^. In addition, a weak coupling anisotropic algorithm with a relaxation time of 1 ps^−1^ was applied to keep a constant pressure. Snapshots were saved every 2 fs. The detailed workflow of the simulations was detailed in Extended Data Figure 7-1*C*.

### Free energy calculation

The free energy of binding between the odorant and the OR was evaluated using the MM-GBSA method, according the following equation:

(1) $$\Delta G_{binding}=〈\Delta G_{complex}〉-〈\Delta G_{OR}〉-〈\Delta G_{odorant}〉.$$

The <> corresponds to an average of a given value extracted from the MD trajectory.

Each term of the Equation 1 can be written as:

(2) $$\Delta G_{X}=\Delta G_{MM}^{{}^{\circ}}\;+\;\Delta G_{solv}-T\Delta S,$$

where X corresponds to the complex, the odorant or the OR. The three terms were the gas-phase contribution to the binding energy, the solvation free energy on binding and an entropic contribution, respectively. The gas-phase contribution was the sum of three terms:

(3) $$\Delta G_{MM}^{{}^{\circ}}=\Delta E_{intra}\;+\;\Delta E_{elec}\;+\;\Delta E_{vdW}.$$

The first term (Δ*E*_intra_) was the difference in internal energy (bond, angle, and dihedral energy) of the OR and the odorant between the bound and unbound forms. The two other terms, (Δ*E*_elec_) and (Δ*E*_vdw_), were the non-bonded electrostatic and van der Waals energies between the bound and unbound form.

The solvation free energy includes two terms:

(4) $$\Delta G_{solv}=\Delta G_{pol}\;+\;\Delta G_{nonpol}.$$

The first terms ΔG_pol_ was estimated using the Generalised Born model of Onufriev, Bashford, and Case. The value of the external dielectric constant was set to 80, while the internal one was set to 1. The non-polar term was considered as the solvent accessible surface area (SASA). The terms ΔG°_MM_ + ΔG_solv._ represent the ΔG_MM-GBSA_ component of the free energy of binding. The MM-GBSA analysis was performed on 40 snapshots sampled every 10 ns and covering the whole molecular dynamics simulation.

## Results

To understand how mammalian ORs encode odorant concentrations, we conducted high-throughput *in vivo* screens via phosphorylated ribosomal protein S6 immunoprecipitation followed by RNA-Seq (pS6-IP-Seq). This characterized comprehensive OR responses and changes in OR activation patterns against ACT and TMT (Fig. 2*A*) from the starting concentration of 0.01% (v/v) to undiluted 100% (v/v).

10.1523/ENEURO.0103-19.2019.f2-2 Extended Data Figure 2-2. Differential expression data for 0.01% ACT. Download Figure 2-2, XLSX file.

**Figure 3.**
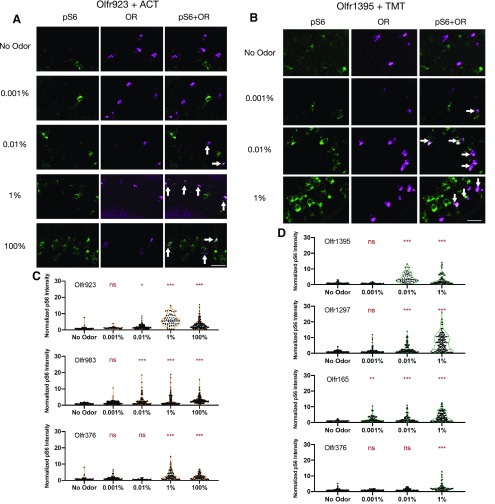
pS6-IP-Seq enrichment data were verified via staining. ***A***, ***B***, Fluorescence microscopy images showing double-label *in situ* of Olfr923 and Olfr1395 OSNs. Green is antibody staining for pS6, magenta is the DIG probe signal. Colocalizations are marked by white arrows, representing odorant activated OSNs. Scale bar: 25 μm. ***C***, ***D***, Violin plots showing the normalized pS6 intensity for DIG-labeled OSNs based on colocalization, each dot represents a probe labeled OSN. Orange and green colors outline ACT and TMT violin plots, respectively. Quantification of the staining data reveals significant difference in normalized pS6 intensity as a result of odorant stimulation; ns, not significant; *p < 0.05, **p < 0.01, ***p < 0.001 by one-way ANOVA, Dunnett’s *post hoc* test (various odorant concentrations are compared with no odor controls) for each probe. *N* = 1–3 mice. See Extended Data Figures 3-1, 3-2 for additional colocalization images and violin plots for ACT responsive ORs. See Extended Data Figures 3-3, 3-4 for lists of pS6-IP-Seq-enriched ORs supporting this figure.

10.1523/ENEURO.0103-19.2019.f2-3 Extended Data Figure 2-3. Differential expression data for 0.1% ACT. Download Figure 2-3, XLSX file.

**Figure 4.**
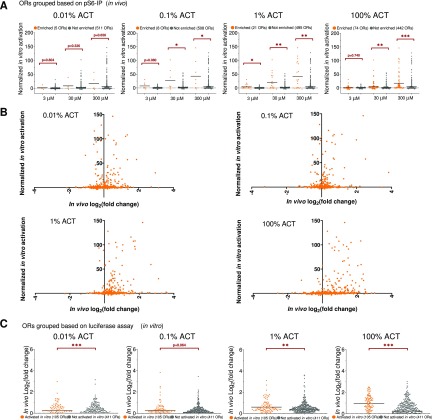
*In vivo* OR enrichment correlates with the *in vitro* activation data. ***A***, *In vitro* Hana3A cell lines cAMP-mediated reporter data for 516 out of the 1088 pS6-IP-Seq mapped intact ORs are classified into *in vivo* pS6-IP-Seq ACT enriched (orange, FDR corrected *p* < 0.05 and log_2_ fold change > 0) and not enriched groups (gray, FDR corrected *p* > 0.05 or log_2_ fold change < 0). *In vitro* normalized fold increase in luciferase signals were plotted for each OR groups across four ACT concentrations; 100% is based on the fold of increase of Olfr1126 activated by 300 μM ACT; 0% is determined by the fold of increase of empty rho-pCI vector stimulated with 3 μM ACT. Black bars represent mean; *p < 0.05, **p < 0.01, ***p < 0.001 by Mann–Whitney test (*in vivo* enriched ORs compared with not enriched ORs). ***B***, Individual pS6-IP-Seq ORs at 0.01%, 0.1%, 1%, and 100% starting concentrations of ACT have their FDR corrected *p* values plotted against *in vitro* activation data. Green dotted line indicates FDR corrected *p* = 0.05 line (ORs to the right have FDR corrected *p* < 0.05, ORs to the left have FDR corrected *p* > 0.05). Red and blue lines indicate mean *in vitro* activation values for *in vivo* enriched and not enriched ORs. ***C***, *In vivo* pS6-IP-Seq fold enrichment was plotted for each OR groups across four ACT concentrations. ORs are classified based on *in vitro* cAMP-mediated reporter data into activated (orange, fold luciferase induction at 300 μM ACT > 2.33) and not activated groups (gray, fold luciferase induction at 300 μM ACT < 2.33). Black bars represent mean; **p < 0.01, ***p < 0.001 by Mann–Whitney test (*in vitro* enriched ORs compared with not enriched ORs). See Extended Data Figures 4-1, 4-2 for *in vitro* data supporting this figure.

10.1523/ENEURO.0103-19.2019.f2-4 Extended Data Figure 2-4. Differential expression data for 1% ACT. Download Figure 2-4, XLSX file.

**Figure 5.**
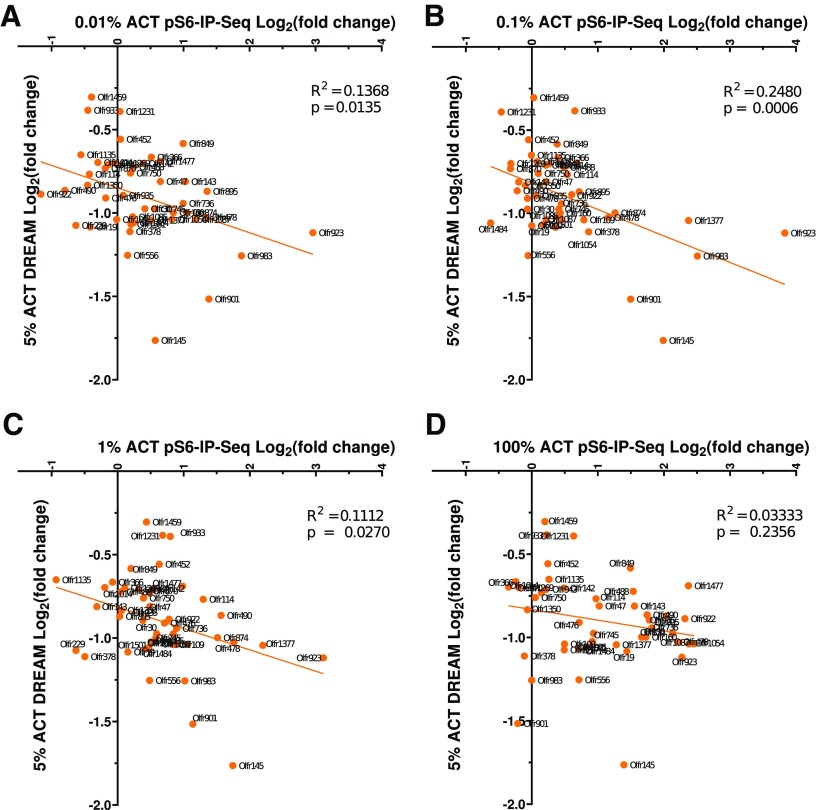
Correlation of pS6 induction and downregulation of mRNA expression. Individual ORs pS6-IP-Seq fold changes based on 0.01% (A), 0.1% (B), 1% (C), and 100% (D) ACT are compared with the DREAM 5% ACT fold changes based on 0.01%, 0.1%, 1%, and 100% ACT are compared with the DREAM 5% ACT fold changes (sequence data realigned using the updated UTRs with coding exons annotation and STAR bioinformatics pipeline). All 45 of the previously reported 5% ACT DREAM responsive ORs were shown. *R*^2^ and *p* values are based on linear regression. See Extended Data Figure 5-1 for the pS6-IP-Seq response of specific DREAM ACT ORs. See Extended Data Figures 5-2, 5-3 DREAM differential expression data supporting this figure.

10.1523/ENEURO.0103-19.2019.f2-5 Extended Data Figure 2-5. Differential expression data for 100% ACT. Download Figure 2-5, XLSX file.

**Figure 6.**
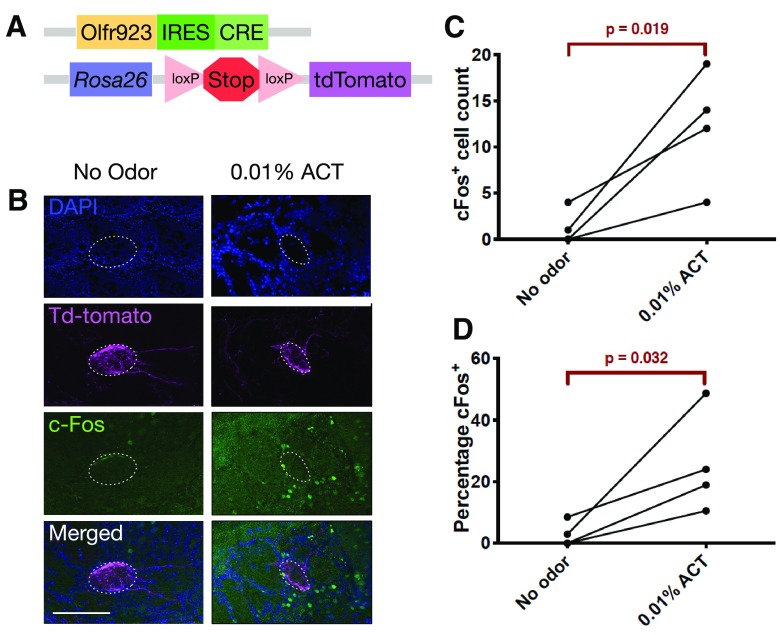
Exposure to 0.01% starting concentration of ACT induces c-Fos expression in Olfr923 periglomerular neurons. ***A***, Schematic drawing of the Olfr923-Cre transgenic mouse targeting vector. ***B***, Fluorescence microscopy image showing the Olfr923 mouse glomerulus. Magenta is Olfr923 labeled by td-tomato, green is periglomerular neurons stained for c-Fos, blue is DAPI nuclear staining. Scale bar: 100 μm. Tissue sections with the greatest density of td-tomato fibers at the glomeruli were used for staining. ***C***, c-Fos-positive periglomerular cell counts around the Olfr923 glomeruli. ***D***, Percentage of c-Fos-positive periglomerular cells, calculated by the number of c-Fos-positive over the total number of DAPI-labeled Olfr923 periglomerular cells. ***C***, *p* values are based on paired *t* test, one-tailed (no odor compared with 0.01% ACT). *N* = 4 mice were used for each odorant condition. Dots connected with a horizontal line represent samples that were stained on the same day. See Extended Data Figures 6-1, 6-2 for the detailed targeting vector construct and additional staining images at the Olfr923 glomeruli.

10.1523/ENEURO.0103-19.2019.f2-6 Extended Data Figure 2-6. Differential expression data for 0.01% TMT. Download Figure 2-6, XLSX file.

**Figure 7.**
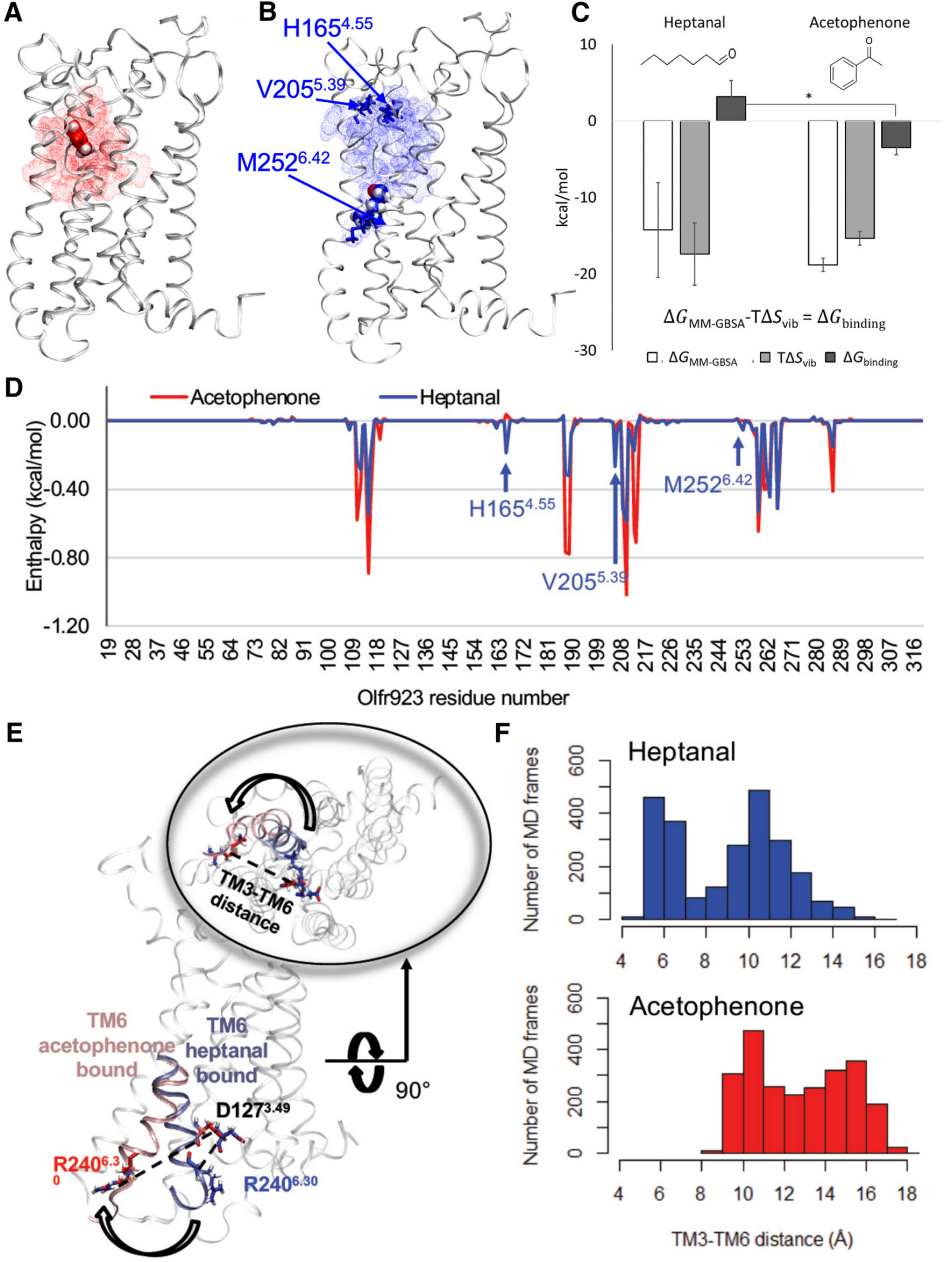
*In silico* investigation of ACT binding to olfr923. The three-dimensional structure of Olfr923 was built by homology modeling and bound to ACT (red, ***A***) and heptanal (blue, ***B***). Helices are represented in white ribbons and the odorant molecules are shown in Van der Walls volumes. The residues interacting with the odorant molecule during molecular dynamics simulations are highlighted by dotted clouds in the corresponding color (ACT-red, heptanal-blue). Residues specific to heptanal binding, H165^4.55^, V205^5.39^ and M252^6.42^ (Ballesteros–Weinstein numbering shown in superscript; Ballesteros and Weinstein, 1995), are represented in blue licorice. ***C***, Computed enthalpy ΔG_MM-GBSA,_ entropy TΔS_vib_, and free energy of binding ΔG_binding_ for heptanal and ACT bound to Olfr923; *p < 0.05 by paired *t* test (ACT compared with heptanal). *N* = 3 simulations per odorant. ***D***, Decomposition per residue of the enthalpy ΔG_MM-GBSA,_ for ACT (red) and heptanal (blue). ***E***, Tridimensional structure of olfr923 in active and inactive conformations. The intracellular part of TM6 and the two residues involved in the ionic lock are highlighted for the ACT bound system (shade of red) and heptanal bound system (blue). Insert, Intracellular view of the receptor. The TM3-TM6 distance monitored in ***F*** is shown by a dotted line. ***F***, Distribution of the number of frames in the molecular dynamics simulations sampling TM3-TM6 distances for ACT (red) and heptanal (blue). See Extended Data Figure 7-1 for GloSensor assay in vitro validation and the molecular dynamics workflow.

10.1523/ENEURO.0103-19.2019.f2-7 Extended Data Figure 2-7. Differential expression data for 0.1% TMT. Download Figure 2-7, XLSX file.

10.1523/ENEURO.0103-19.2019.f2-8 Extended Data Figure 2-8. Differential expression data for 1% TMT. Download Figure 2-8, XLSX file.

10.1523/ENEURO.0103-19.2019.f2-9 Extended Data Figure 2-9. Differential expression data for 100% TMT. Download Figure 2-9, XLSX file.

10.1523/ENEURO.0103-19.2019.f2-10 Extended Data Figure 2-10. Differential expression data for dorsal ventral RNA-Seq. Download Figure 2-10, XLSX file.

10.1523/ENEURO.0103-19.2019.f2-11 Extended Data Figure 2-11. ACT responsive ORs across each odorant concentration. Download Figure 2-11, XLSX file.

10.1523/ENEURO.0103-19.2019.f2-12 Extended Data Figure 2-12. TMT responsive ORs across each odorant concentration. Download Figure 2-12, XLSX file.

### Modified pS6-IP-Seq enriched more ORs

We updated the pS6-IP-Seq method as originally described by Jiang et al. (2015), in an effort to profile ORs expressed in active OSNs that respond to lower concentrations of odorants. Figure 1*A* shows the updated pS6-IP-Seq pipeline. Similar to the Jiang et al. (2015) publication, we habituated and stimulated a mouse for 1 h with 10 μl of odorant at a given starting odorant concentration. We replaced the polyclonal antibody against pS6 with a monoclonal alternative to improve immunoprecipitation consistency. We updated annotations of OR genes to also include UTRs containing Bowtie aligned transcripts (Langmead et al., 2009; Li et al., 2009). As an example, Figure 1*B* shows coding region (CDS) annotation and updated annotation (CDS + UTR) for an ACT OR, Olfr923. Figure 1*C* also shows Olfr923’s transcripts read depth aligned with STAR based on the new OR annotation (for details, see Materials and Methods; Dobin et al., 2013; Bray et al., 2016).

The updated OR annotations now include 1385 UTR annotated ORs, largely based on the work of Ibarra-Soria et al. (2014). To complete this set of OR definitions, we made three additional modifications: reannotation of 58 coding exonal ORs genes to include noncoding UTRs, correction of a misannotated OR (Olfr151) and addition of four previously unannotated ORs from the refGene database (O'Leary et al., 2016). More ORs were significantly enriched (FDR corrected *p* < 0.05) at 1% and 100% ACT and TMT with the UTRs included gene annotations, compared with ORs reported by Jiang et al. (2015), suggesting a more sensitive detection of OR enrichment with the updated pS6-IP-Seq method (Fig. 2*B*,*D*; Extended Data Figs. 2-11, 4-2).

### OR repertoires with different odorant concentrations

An increase in the number of ORs suggests that the updated pS6-IP-Seq method may allow us to identify activated ORs at lower odorant concentrations than what has been previously reported (Jiang et al., 2015). To test this, we stimulated mice with 0.01%, 0.1%, 1%, and 100% (v/v) of ACT and performed the updated pS6-IP-Seq at each condition (*n* = 3; for details, see Materials and Methods). At the lowest starting concentration, 0.01%, ACT enriched six ORs (FDR corrected *p* < 0.05; Fig. 2*B*). The number of ORs recruited increased from six to 115 (Fig. 2*B*) when the starting odorant concentration was increased from 0.01% to 100%. This shows that more ORs were activated at higher concentrations of odorants (Extended Data Fig. 2-11). Trace amine associated receptors (Taars), a small family of chemosensory receptors activated by volatile amines (Liberles and Buck, 2006) were not significantly enriched by the tested odorants.

To investigate the phylogenetic relationship of ORs responding to an odorant, we plotted enriched ORs on protein sequence-based phylogenetic trees (Fig. 2*C*). As the odorant concentrations increase, more diverse sets of ORs were enriched (Fig. 2*C*), consistent with findings previously reported (Jiang et al., 2015).

We performed the same experiment and analyses for another odorant, TMT. The number of ORs recruited increased from three to 68 for TMT (Fig. 2*D*) when the starting odorant concentration was increased from 0.01% to 100% TMT (Extended Data Fig. 3-3). Similar to ACT, more diverse sets of ORs are enriched as the odorant concentrations increase (Fig. 2*E*). These findings are consistent with earlier studies based on glomerular activation in the olfactory bulb (Rubin and Katz, 1999; Wachowiak and Cohen, 2001; Fried et al., 2002; Wilson et al., 2017) and complement these studies by identifying ORs that respond to a wide range of concentrations of odorant.

The distribution of OSNs expressing a given OR is confined within continuous and partially overlapping spatial zones along the dorsomedial (dorsal)–ventrolateral (ventral) axis on the olfactory epithelium (Ressler et al., 1993; Vassar et al., 1993; Miyamichi et al., 2005; Tan and Xie, 2018). To test whether active ORs for each odorant show bias in their zonal distributions, we conducted an RNA-Seq using dorsal and ventral olfactory mucosa (for details, see Materials and Methods) and found 412 and 558 ORs to be significantly enriched in dorsal and ventral zones, respectively (Fig. 2*F*; Extended Data Fig. 2-10). Our RNA-Seq data classifying the ORs into dorsal and ventral zones (Extended Data Fig. 2-1*A*^b^) (Table 1) is consistent with the zone index data reported by Tan and Xie (2018; *p*^b^ < 0.001, Mann–Whitney test). Comparing between the distribution of responsive dorsal and ventral ORs of ACT and TMT at different concentrations, we found that ACT enriched ORs as a group are more dorsally distributed than TMT enriched ORs from low to high odorant concentrations (Fig. 2*F*^c^; Extended Data Fig. 2-1*B*^d^) (Table 1), suggesting that biased zonal populations of ORs are responding to each of the tested odorants.

### Most sensitive ORs may not be most robust responders at higher concentrations

At 0.01% starting concentration of ACT, Olfr923 was the most significantly enriched OR with the largest log_2_ fold change (Log_2_FC) amongst significantly enriched ORs (FDR corrected *p*^a^ = 1.56 × 10^−13^, Log_2_FC = 2.96; Fig. 2*B*), suggesting it is among the most sensitive ORs that recognize ACT. Olfr923 remained significantly enriched from 0.1% to 100% ACT (FDR corrected *p* value *p*^a^ < 0.001; Fig. 2*B*). However, the rank order of Olfr923 in terms of FDR corrected *p* values and Log_2_FCs went down at 1% and higher concentrations of ACT (Fig. 2*B*). Olfr983 is the second most significantly enriched OR with the second largest Log_2_FC amongst significantly enriched ORs at 0.01% ACT (FDR corrected *p*^a^ = 2.55 × 10^−9^, Log_2_FC = 1.88; Fig. 2*B*). Similarly, the rank order of Olfr983’s activation also went down at higher starting concentrations of ACT (Fig. 2*B*). In contrast, Olfr907 and Olfr898, ORs with the largest and second largest Log_2_FCs among significantly enriched ORs at 100% ACT, were not significantly enriched at 1% and lower starting concentrations of ACT (Fig. 2*B*). Olfr30, the most significantly enriched OR at 100% ACT, was also not significantly enriched at 1% and lower starting concentrations of ACT. In addition, Olfr376, the most significantly enriched OR at 1% ACT, was also not significantly enriched at 0.1% and lower concentrations of ACT (Extended Data Fig. 3-3). These results suggest that sensitive ORs responding at lower odorant concentrations may not be the most robust responders at higher odorant concentrations, and vice versa.

We observed similar trends with TMT. The rank order of three significantly enriched ORs at 0.01% TMT (Olfr1395, Olfr165, and Olfr1297) also went down in terms of FDR corrected *p* values and Log_2_FCs at 1% and 100% starting concentrations of TMT (Fig. 2*D*). In contrast, Olfr837 and Olfr506, ORs with the largest and the second largest Log_2_FCs among ORs significantly enriched ORs at 100% TMT, were not significantly enriched at 0.1% and 0.01% TMT (Fig. 2*D*). Lastly, Olfr531 and Olfr376, the most and the second most significantly enriched OR at 100% TMT, were not significantly enriched at 0.01% (Fig. 2*D*; Extended Data Fig. 3-4).

To validate pS6 induction for OSNs in a set of significantly enriched ORs, we conducted *in situ* hybridization and pS6 immunostaining for ORs responsive at 0.01% starting odorant concentrations. Consistent with our pS6-IP-Seq data, both Olfr923 and Olfr983, but not Olfr376-expressing OSNs (serving as a control) showed significantly higher pS6 signals at 0.01% ACT compared with no odor controls (Fig. 3*A*,*C*^e^; Extended Data Fig. 3-1) (Table 1). Violin plots for Olfr1427 and Olfr901 (pS6-IP-Seq enriched at 0.01% ACT) show significantly higher pS6 signals in staining at 1% and higher starting ACT concentrations (Extended Data Fig. 3-2^f^). *In situ* hybridization and pS6 immunostaining also confirmed the activation of Olfr1395, Olfr1297, and Olfr165-expressing OSNs towards TMT (Fig. 3*B*,*D*^g^; Extended Data Fig. 3-1) (Table 1). Consistent with our pS6-IP-Seq data, Olfr376-expressing OSNs did not show significantly higher pS6 signals at 0.01% starting concentration of TMT (Fig. 3*D*^g^; Extended Data Fig. 3-1) (Table 1). As a negative control, we also stained for the pS6 intensity of Olfr1395 when mice were exposed to ACT instead of TMT. Consistent with our pS6-IP-Seq’s differential expression data for ACT, Olfr1395 did not show significantly higher pS6 signals at any tested ACT concentrations (Extended Data Fig. 3-2^f^) (Table 1). Together, our data verified a set of sensitive ORs for ACT and TMT.

10.1523/ENEURO.0103-19.2019.f3-1 Extended Data Figure 3-1 Fluorescence microscopy images showing double-label *in situ* and immunostaining of OSNs. Magenta is the DIG OR probe signal, green is antibody staining for pS6. Colocalizations are marked by white arrows. Colocalization is observed at starting concentrations of 0.01% odorant and above. Scale bar: 25 μm. Download Figure 3-1, EPS file.

10.1523/ENEURO.0103-19.2019.f3-2 Extended Data Figure 3-2 Violin plots showing the normalized pS6 intensity for ACT stimulated (orange outline) DIG-labeled OSNs at the mice olfactory epithelium. Olfr1427 and Olfr901 were ORs enriched by 0.01% ACT based on pS6-IP-Seq. Olfr1395 (significantly enriched by TMT based on pS6-IP-Seq) serves as a negative control for ACT; **p < 0.01, ***p < 0.001 by one-way ANOVA, Dunnett’s *post hoc* test (various starting odorant concentrations are compared to no odor controls) for each probe. *N* = 1–3 mice. Download Figure 3-2, EPS file.

10.1523/ENEURO.0103-19.2019.f3-3 Extended Data Figure 3-3. 138 pS6-IP-Seq ACT enriched ORs. Download Figure 3-3, XLSX file.

10.1523/ENEURO.0103-19.2019.f3-4 Extended Data Figure 3-4. 80 pS6-IP-Seq TMT enriched ORs. Download Figure 3-4, XLSX file.

### Correlation between pS6-IP-Seq and *in vitro* or responses

The *in vitro* responses of a panel of 500 ORs against ACT based on cAMP-mediated luciferase reporter gene assays in heterologous cells were reported to be correlated with the enrichment of ORs from pS6-IP-Seq (Jiang et al., 2015). Here we compared our new *in vivo* ACT pS6-IP-Seq dataset to the *in vitro* ACT dataset reported by Jiang et al. (2015). We first investigated whether significantly enriched ORs grouped using *in vivo* pS6-IP-Seq (orange circles) showed higher *in vitro* activation at three different concentrations of ACT (3 μM, 30 μM, and 300 μM), compared with the “not *in vivo* enriched” OR group (Fig. 4*A*, gray circles). Additionally, we created a graph for each ACT concentration, with FDR corrected *p* values on the *x*-axis, and in vitro responses on the *y*-axis to provide a sense how FDR corrected *p* values correlates with in vitro responses (Fig. 4*B*). Overall, 89 out of the 138 *in vivo* enriched ORs were tested in vitro and 46 (52%) were activated (Extended Data Fig. 4-1). Consistent with the results of Jiang et al. (2015), we found that the *in vivo* enriched OR group has a significantly higher *in vitro* activation from 0.1% to 100% ACT, but there are instances where *in vivo* enriched ORs do not show *in vitro* responses, and vice versa.

10.1523/ENEURO.0103-19.2019.f4-1 Extended Data Figure 4-1 *In vitro* versus in vivo response data. Download Figure 4-1, XLSX file.

10.1523/ENEURO.0103-19.2019.f4-2 Extended Data Figure 4-2 Comparison across Jiang et al. (2015)’s 86 responsive ACT OR + Olfr888. Download Figure 4-2, XLSX file.

Similarly, we observed that the ORs grouped based on *in vitro* luciferase assay activation (orange circles) had a significantly higher pS6-IP-Seq fold enrichment at all the ACT concentrations tested, compared with the “not *in vitro* activated” OR group (Fig. 4*B* ^h^, gray circles) (Table 1); 46 out of the 105 *in vitro* activated ORs were also enriched *in vivo* based on pS6-IP-Seq (Extended Data Fig. 4-1).

### Evaluating pS6-IP-Seq versus DREAM techniques

The DREAM technique is another method for the *in vivo* identification of odor activated ORs. DREAM relies on the transcriptional downregulation of individual ORs after ∼5 h of odor exposure (von der Weid et al., 2015). Using DREAM, von der Weid et al. (2015) identified a set of ORs, 22 of which were confirmed by qPCR, to be responsive at 5% ACT.

To understand the relationship between DREAM and our updated pS6-IP-Seq, we focused on the top 45 ORs whose mRNA expressions were downregulated by 5% ACT (von der Weid et al., 2015; Extended Data Fig. 5-2). We remapped the sequence data using our updated OR definitions and performed differential expression analysis using the same bioinformatics pipeline as our pS6-IP-Seq datasets (Extended Data Fig. 5-3; for details, see Materials and Methods). Consistent with the von der Weid et al. (2015) study, all the top 45 ORs were downregulated. Comparing the DREAM data with our pS6-IP-Seq data, many of the top 45 ORs showed positive Log_2_FC values in pS6-IP-Seq (Fig. 5; Extended Data Fig. 5-1). Overall, 19 of the top 45 ORs showed significant enrichment (i.e., FDR corrected *p* < 0.05) at least in one of the tested concentrations in our pS6-IP-Seq data. Log_2_FC values were negatively correlated between DREAM at 5% ACT and pS6-IP-Seq from 0.01% to 1% ACT (*p* values < 0.05 and *R*^2^ range from 0.11 0.25; Fig. 5*A* ^i^*–C*^i^) (Table 1), while Log_2_FC values were weakly correlated between DREAM at 5% ACT and pS6-IP-Seq at 100% ACT (*p* = 0.2356 and *R*^2^ = 0.03; Fig. 5*D* ^i^) (Table 1). Altogether, our analysis suggests that the degrees of pS6 induction and downregulation of mRNA expression are moderately correlated.

10.1523/ENEURO.0103-19.2019.f5-1 Extended Data Figure 5-1 Volcano plots showing our pS6-IP-Seq data for the 26 ACT ORs found to be differentially expressed solely in the DREAM technique. Log_2_ fold change (logFC) and FDR corrected *p* values are shown based for 1% and 100% ACT. Download Figure 5-1, EPS file.

10.1523/ENEURO.0103-19.2019.f5-2 Extended Data Figure 5-2 Comparison between DREAM and pS6-IP-Seq data. Download Figure 5-2, XLSX file.

10.1523/ENEURO.0103-19.2019.f5-3 Extended Data Figure 5-3 Differential expression data based on von der Weid et al. (2015)’s DREAM data. Download Figure 5-3, XLSX file.

### Validating Olfr923 with a gene knock-in approach

As an independent approach to demonstrate *in vivo* activation of Olfr923 expressing OSNs by ACT, we generated IRES-Cre gene knock-in mice at the Olfr923 locus and crossed them with Rosa26-lox-stop-lox-tdTomato reporter mice (Fig. 6*A*; Extended Data Fig. 6-1) to label the Olfr923 glomerulus (Madisen et al., 2010).

10.1523/ENEURO.0103-19.2019.f6-1 Extended Data Figure 6-1 *A*, Targeting vector construction through homologous recombination mediated BAC recombineering. ***B***, Schematic of the Olfr923-IRES-Cre targeting. ***C***, Targeting vector map. Download Figure 6-1, EPS file.

10.1523/ENEURO.0103-19.2019.f6-2 Extended Data Figure 6-2 *A*, Fluorescence microscopy images of the olfactory bulb. Blue is DAPI nuclear staining, magenta is Olfr923 labeled by td-tomato, green is periglomerular neurons stained for c-Fos. Merged images show periglomerular c-Fos activity around the Olfr923 glomeruli. Scale bar: 100 μm. ***B***, Individual quantification of the c-Fos activity for each glomerulus, quantified areas are shown in as cropped glomeruli images on the left. Dots connected with a horizontal line represent samples that were stained on the same day. Download Figure 6-2, EPS file.

To ask whether the Olfr923 glomeruli in the olfactory bulb were activated, we conducted c-Fos immunostaining in periglomerular cells induced by odor stimulation (Guthrie et al., 1993). Mice exposed to 0.01% starting concentration of ACT displayed a significant increase in c-Fos induction in periglomerular cells surrounding the Olfr923 glomeruli compared with no odor controls both in terms of cell counts and percentages of c-Fos-positive cells (*p*^j^ < 0.05 paired *t* test, one-tailed; Fig. 6*B*,*C*; Extended Data Fig. 6-2), suggesting ACT activates Olfr923 OSNs and also the Olfr923 glomeruli.

### The free energy of binding shows the high affinity of Olfr923 for ACT

Our *in vivo* and *in situ* data consistently showed ACT to be an activator for Olfr923. Lastly, we asked if Olfr923 has evidence of structural properties that are favorable for ACT binding. Heptanal is a non-agonist of Olfr923 as previously reported (Ibarra-Soria et al., 2017). We first aimed to confirm Olfr923 activation *in vitro* using the GloSensor assay system to monitor the real time response of Olfr923 against ACT and heptanal. ACT or heptanal was dissolved into medium and odor-mediated cAMP induction was monitored in real time. Increases of over time were observed when Olfr923 was exposed to ACT, an indication of Olfr923 activation by ACT, while no significant luminescence increase was observed when Olfr923 was exposed to heptanal (Extended Data Fig. 7-1*A*). To quantify Olfr923 activations, we analyzed the area under the curve (AUC) of the normalized luminescence and generated dose response curves, confirming differential activation of Olfr923 by ACT and heptanal (Extended Data Fig. 7-1*B*).

A three-dimensional model of Olfr923 was built by homology modeling (for details, see Materials and Methods). ACT was docked into the canonical binding cavity of Olfr923, and heptanal serves as a negative control. Heptanal is a non-agonist of Olfr923 as previously reported (Ibarra-Soria et al., 2017). This complex was embedded in a membrane model, solvated in water and submitted to multiple molecular dynamics simulations. After several steps of minimization, equilibration and 800 ns of production with no constraints, ACT remained closely associated with the canonical binding cavity (Fig. 7*A*). The odorant binding cavity is more constricted around ACT, as shown for other non-olfactory GPCR structure bound to agonist (Wingler et al., 2019). However, heptanal exited the binding cavity to enter the lipid bilayer that composes the membrane, suggesting a low ligand-OR binding cavity affinity (Fig. 7*B*). Next, we computed the free energy of binding (ΔG_binding_) by MMGBSA calculation (Topin et al., 2014; Fig. 7*C*). This method was previously shown to be effective in discriminating OR agonists from non-agonists (Topin et al., 2014). Olfr923 bound to ACT has a favorable negative ΔG_binding_ (Fig. 7*C*). In contrast, heptanal has an unfavorable positive ΔG_binding_ (*p*^k^ = 0.0371 paired *t* test, one-tailed; Fig. 7*C*).

10.1523/ENEURO.0103-19.2019.f7-1 Extended Data Figure 7-1 *A*, Liquid-phase ACT and heptanal stimulation of Olfr923 with the GloSensor assay system. Luminescence in each well was measured once every 90 s for six cycles, and the values were normalized with the initial value for Olfr923-pCI and response of the pCI control at the given time point. *N* = 3 wells were used for each concentration. Error bars indicate SEM. ***B***, AUC indicates the sum of normalized luminescence (normalized to pCI vector+ buffer condition) from initial response to final response. Error bars indicate SEM. ***C***, Molecular dynamics workflow. Download Figure 7-1, EPS file.

In order to identify the residues of Olfr923 involved in the stabilization of ACT and destabilization of heptanal, we decomposed the enthalpy of binding (ΔG_MM-GBSA_) by the contribution of each residue (Fig. 7*D*). We saw that ACT binding is more strongly stabilized than heptanal with lower enthalpy values for its interaction with residues of the Olfr923 binding cavity. However, residues H165^4.55^, V205^5.39^, and M252^6.42^ (Ballesteros–Weinstein numbering is shown in superscript; Ballesteros and Weinstein, 1995) stabilized heptanal binding more than for ACT in our molecular dynamics simulations (Fig. 7*D*). To identify the positions of the residues involved in the binding of both molecules, we projected them on our three-dimensional model (Fig. 7*A*,*B*). ACT engaged in interactions exclusively with residues belonging to the canonical odorant binding cavity of Olfr923 (Man et al., 2004; Katada et al., 2005; de March et al., 2015a), positioning ACT in an effective binding position likely to trigger the receptor activation (Fig. 7*A*). Heptanal was more mobile into the binding cavity. Interestingly, the residues H165^4.55^, V205^5.39^, and M252^6.42^ which participated only in heptanal stabilization are always located outside of the canonical binding cavity (Fig. 7*B*,*D*), either in the extracellular part of the receptor or between TM4 and TM5 under the canonical binding cavity.

This explains the tendency of heptanal to exit the canonical binding cavity during our molecular dynamic simulations. Furthermore, it was shown recently that molecular dynamic simulations of ORs can sample active or inactive states of the receptor when bound to an agonist or a non-agonist, respectively (de March et al., 2015b, 2018). The most accurate parameter to monitor receptor activation in molecular dynamics simulations is the distance between the intracellular part of TM3 and TM6, particularly between the two protagonists of the ionic lock in GPCR (Fig. 7*E*; Altenbach et al., 2008; de March et al., 2015b, 2018). The inactive conformation of an OR is maintained by the ionic interaction, called ionic lock, between D^3.49^ belonging to the motif **D^3.49^**RY common to the GPCR superfamily and R^6.30^ belonging to the motif R^6.30^xKAFSTCASH specific to the OR family. During activation, conformational changes in the receptor are associated with a break of this ionic interaction and a switch of the intracellular part of TM6 toward the membrane, opening an intracellular cavity for the G-protein coupling (de March et al., 2015b, 2018). By observing our multiple molecular dynamics simulations, we noticed that Olfr923 structures bound to ACT show mostly opened ionic locks (>12 Å), suggesting active conformation (Fig. 7*E*,*F*). However, Olfr923 structures bound to heptanal exhibit a majority of receptor conformations possessing a close ionic lock (<12 Å), suggesting that the Olfr923 is mainly in its inactive conformation (Fig. 7*E*,*F*). This difference in the distribution of receptor conformations between ACT and heptanal shows that our model captured the ligand binding features required to trigger Olfr923 activation. Altogether, our data suggests that the Olfr923 binding cavity has favorable properties for ACT effective binding, and further supports our findings that ACT binds to and activates Olfr923.

Our results from *in vivo*, *in situ*, and *in silico* approaches, together with the reported activation of Olr923 by ACT *in vitro* (Jiang et al., 2015), suggest that ACT binds and activates Olfr923.

## Discussion

In this study, we investigate how odorant concentration information is represented at the receptor level. This comprehensive representation is reported as activated OR across a 10,000-fold starting concentration range. We identified some of the most sensitive ORs for ACT and TMT and validated the activation of Olfr923 by ACT at the glomerular level in the olfactory bulb.

### Updated method to identify ORs activated by odors *in vivo*

To more accurately describe the concentration-dependent combinatorial OR code, we report an updated pS6-IP-Seq method to profile mRNA expression in odor activated OSNs. We used a monoclonal pS6 antibody, streamlined the library preparation methods (Knight et al., 2012; Jiang et al., 2015), and adopted STAR for efficient sequence reads mapping (Dobin et al., 2013). We can now identify more ORs at given odor concentrations and at lower and more physiologically relevant odor concentrations. The updated pS6-IP-Seq can be adopted to identify responsive ORs across a wide range of monomolecular odorants, odorant mixtures, and complex natural odors to further our understanding of peripheral odor coding (Isogai et al., 2018).

Our OR lists overlap with a majority of the ORs identified by the Jiang et al. (2015) publication. Out of the total 86 ACT responsive ORs reported by Jiang et al. (2015), 51 of these ORs were also identified by our current study. The vast majority of the non-overlapping ORs exhibit a positive Log_2_FC, indicating that these ORs were enriched but did not reach our statistical criteria (FDR corrected *p* < 0.05). It is also likely that the difference in antibodies against pS6 (i.e., polyclonal antiserum in Jiang et al. vs monoclonal antibodies in the current study) contribute some differences (Jiang et al., 2015). Changes in the bioinformatics pipelines also contribute to differences.

Our data support the notion that higher concentration of odorants activates more ORs (Fried et al., 2002; Mainland et al., 2014). In the vast majority of cases, ORs significantly enriched with odor stimulation at a low concentration are also enriched at higher concentrations. However, there are some instances where ORs show significant enrichment only at lower concentrations. The statistical criteria based on the FDR corrected *p* < 0.05 cutoff does not represent the critical boundary between responsive ORs and non-responsive ORs. It serves as a selection criterion to identify populations of responsive ORs activated backed by statistical confidence. It is also likely that the proportional nature of RNA-Seq contributes to apparent lower fold changes at the higher odorant concentration. With future advancements in next-generation sequencing data collection and analysis, we expect refinement in the list of enriched ORs.

### OR populations encoding odor concentrations

Humans perceive the same odor with higher intensity and varying degree of pleasantness as odor concentration increases (Moskowitz et al., 1976; Anderson et al., 2003). In some cases, odor quality and/or pleasantness shift when different concentrations of the same odorant are presented (Gross-Isseroff and Lancet, 1988), although there is no such report for ACT and TMT. Higher concentrations of odorants that result in higher OSN spiking rates (Rospars et al., 2000; Bhandawat et al., 2005; Grosmaitre et al., 2006) are likely to result in higher S6 phosphorylation as shown in the retinal cells (Milner and Do, 2017). This cascade of events, could, in turn, contribute to the neural representation of elevated odor intensity. Higher concentration of odors activate additional, lower affinity ORs at the olfactory epithelium, resulting in more glomerular activations in the olfactory bulb (Fried et al., 2002; Khan et al., 2010). These additionally recruited, low-affinity ORs may drive differences in odor quality and perceived intensity by modulating neuronal firing in higher olfactory areas (Stettler and Axel, 2009). In this study, we identified a set of ORs responding to both low and high concentrations odorant and another set of ORs recruited only at high odorant concentrations. In the future, resources provided in this study will be useful in defining the roles of high-affinity and low-affinity ORs by targeting specific ORs to manipulate in reference to their *in vivo* activation profiles.

### Specific ORs encoding odor identity

Another dimension of smell encoding, besides keeping track of odorant concentrations, is for neurons to also represent concentration differences of a given odor (Parabucki et al., 2019). Despite facing a dynamic environment where they encounter hundreds to thousands of volatile odors at various concentration gradients, humans and animals are capable of maintaining stable odor quality perception across concentrations (Krone et al., 2001; Wachowiak and Cohen, 2001; Uchida and Mainen, 2007; Mainland et al., 2014; Sirotin et al., 2015; Wilson et al., 2017).

What is not yet clear is how odor identities are encoded and maintained despite massive differences in the activated OR repertoires across concentrations. Stable odor ratio information (Uchida and Mainen, 2007), input-output transformation at the level of the olfactory bulb (Storace and Cohen, 2017) and early activated ORs during the first sniff (Wilson et al., 2017) are implicated in concentration invariance. Sensitivity of ORs, as well as abundance and positions of OSNs expressing individual ORs within the olfactory epithelium (zonal expression), can play a role in determining the timing of neuronal activation at the level of glomeruli in the olfactory bulb (Schneider et al., 1966; D’Hulst et al., 2016). Our datasets reporting ORs responding to low concentrations of odorants, in conjunction with a number of OSNs expressing different ORs which correlates with OR mRNA abundance (Ibarra-Soria et al., 2014) together with zonal expression of ORs (Ressler et al., 1993; Vassar et al., 1993; Miyamichi et al., 2005; Tan and Xie, 2018) can provide valuable information in targeting a specific set of ORs to investigate their role in odor coding.

### Comparisons of pS6-IP-Seq, DREAM and heterologous expression in measuring or responses

The DREAM technique identifies odor responsive ORs based on a decrease in OR mRNA abundance after odor stimulation (von der Weid et al., 2015). Our analysis suggests that OR responses based on *in vitro* assays and the two *in vivo*-based assays (pS6-IP-Seq and DREAM) correlated each other yet they did not show strong linear correlations. For example, Olfr923 is one of the most sensitive and robust ORs responding to ACT based on pS6-IP-Seq, ranking top in terms of Log_2_FC amongst significantly enriched ORs from 0.01% to 1% ACT. Olfr923 ranked 36^th^ out of the top 45 responsive ORs based on 5% ACT DREAM RNA-Seq fold change in the von der Weid publication, Olfr923 now ranks fifth when our bioinformatics pipeline is used to reanalyze the 5% ACT DREAM RNA-Seq data (von der Weid et al., 2015; Fig. 5; Extended Data Fig. 5-3). When tested *in vitro* via the luciferase assay, Olfr923 ranked 32nd out of the 105 responsive ORs based on fold luciferase induction with 300 μM ACT (Jiang et al., 2015). Difficulties in functionally expressing certain ORs in heterologous cells partly explains the differences. Future mechanistic understanding of how odorant activation drives S6 phosphorylation or OR mRNA decrease should help explain these differences across the *in vivo* and *in vitro* OR response studies. In addition, the abundance of ORs can also be widely varied (Ibarra-Soria et al., 2014). Some ORs with low read counts did not reach the set significance (FDR corrected *p* > 0.05) despite large fold changes. A deeper sequencing read depth will help determine whether these ORs are bona fide ORs responding to the tested odorants.

In summary, our study comprehensively identifies ORs responding to varying concentrations of odorants and contributes to the understanding of how odorant concentration information is encoded at the receptor level in the peripheral olfactory system. Identifying ORs responding to both low to high odorant concentrations will be valuable in future studies evaluating the contribution of high-affinity and low-affinity ORs towards odor perception.

**Table 1:** Statistical table

Location	Data structure	Type of test	Power/confidence intervals				
**a**	Normal distribution	Differential gene expression analysis	Multiple comparison adjusted FDR *p* values are reported				
**b**	Non-normal distribution	Mann–Whitney test	*p* < 0.0001				
**c**	Categorical	Fisher’s exact test	0.1% odorant *p* = 0.0147; 1% odorant *p* = 0.0021; 100% odorant *p* = 0.0057				
**d**	Non-normal distribution	Mann–Whitney test	0.1% odorant *p* < 0.0001; 1% odorant *p* = 0.0855; 100% odorant *p* = 0.0029				
**e**	Normal distribution	One-way ANOVA followed by Dunnett’s *post hoc* test		Olfr923	Olfr983	Olfr376	
			No odor vs 0.001%	0.884	0.1106	0.5127	
			No odor vs 0.01%	0.0187	<0.0001	0.646	
			No odor vs 1%	<0.0001	<0.0001	<0.0001	
			No odor vs 100%	<0.0001	<0.0001	0.0004	
**f**	Normal distribution	One-way ANOVA followed by Dunnett’s *post hoc* test	Olfr1427: no odor vs 0.001% = 0.9982, no odor vs 0.01% = 0.9106, no odor vs 1% < 0.0001 Olfr901: no odor vs 0.001% = 0.7569, no odor vs 0.01% = 0.0930, no odor vs 1% = 0.0030, no odor vs 100% < 0.0001 Olfr1395: no odor vs 0.001% = 0.9889, no odor vs 0.01% = 0.8116, no odor vs 1% = 0.6882, no odor vs 100% = 0.3402				
**g**	Normal distribution	One-way ANOVA followed by Dunnett’s *post hoc* test		Olfr1395	Olfr1297	Olfr165	Olfr376	
			No odor vs 0.001%	0.811	0.4429	0.0014	0.9326	
			No odor vs 0.01%	<0.0001	<0.0001	0.0009	0.7041	
			No odor vs 1%	<0.0001	<0.0001	<0.0001	<0.0001	
**h**	Non-normal distribution	Mann-Whitney test	Normalized *in vitro* activation data: 0.1% ACT *p*(3μM) = 0.0795, *p*(30μM) = 0.0289, *p*(300μM) = 0.0458; 1% ACT *p*(3μM) = 0.0120, *p*(30μM) = 0.0029, *p*(300μM) = 0.0011; 100% ACT *p*(3μM) = 0.7345, *p*(30μM) = 0.0011, *p*(300μM) < 0.0001; Fold enrichment *in vivo* data: 0.01% ACT *p* < 0.0001; 0.1% ACT *p* = 0.0642; 1% ACT *p* = 0.0023; 100% ACT *p* < 0.0001				
**i**	Normal distribution	Linear regression	Slope deviation from zero *p* values are reported				
**j**	Normal distribution	Paired *t* test	Cell count *p* = 0.019, percentage cFos *p* = 0.032				
**k**	Normal distribution	Paired *t* test	*p* = 0.0371				

## Synthesis

Reviewing Editor: Anna Menini, SISSA (International School of Advances Studies) Decisions are customarily a result of the Reviewing Editor and the peer reviewers coming together and discussing their recommendations until a consensus is reached. When revisions are invited, a fact-based synthesis statement explaining their decision and outlining what is needed to prepare a revision will be listed below.

Synthesis

Both Reviewers highlight the relevance of this work that tested, in vivo, odorant receptors that responded to a given chemical. The Reviewers have specific suggestions for improving the manuscript related to data analysis and interpretation and raised some concerns that must be addressed. In particular, important points of agreement from the online discussion are the following:

1) please add information relative to the identified responsive ORs at different concentrations, as this is at the core of the study and it is critical to compare results.

2) the authors should re-evaluate their comparison to the DREAM technique more rigorously, including using the same standards to compare against prior pS6-IP-Seq data. Even if the authors decide to eliminate their DREAM experiment data, which seems a likely response, a more rigorous comparison of the pS6-IP-Seq data would be helpful.

3) Figure 4: please do add the plots suggested by reviewer #2.

Please also respond to the other comments detailed below and make appropriate revisions to your manuscript.

Reviewer #1

Statements about the concentrations tested need to be worded more accurately. In vivo, the concentrations reaching the ORs are not known, so specific concentrations cannot be stated except as the starting concentrations used to produce varying concentrations experienced by the ORs. It is correct to state that headspace air above specific concentrations were used, but no more than that.

Discussion lacks an explanation for why ORs responding to low concentrations sometimes fail to show responses to higher concentrations in the pS6-IP-Seq method, or show reduced magnitude of response. This apparent discrepancy deserves explanation.

A glaring omission is a lack of tables listing the ORs responsive to ACT and TMT. Best if these tables give the fold-differences and p-values at each concentration.

Also lacking are statements about whether ACT and TMT are considered concentration-invariant odorants in humans and rodents. These statements need to be followed with a few words about whether the data are consistent with concentration invariance.

Do the in vivo approaches tested detect TAAR responses? What do the TAAR data look like? These are significant omissions given the use of amine-containing odorant, TMT.

135. “Though we did not formally test between the two sexes,” Add “for differences”

Fig. 2B and 2D. Readers will assume that n = a number means replicates, not the number of significant ORs. Instead of (n = 3) label this as (3 ORs), (4 ORs), etc.

Also, a statement about the ORs with very large fold-differences that do not reach significance is in order. What explains these data and are they indicative of fundamental problems with the technique?

The same n = a number problem occurs in Fig. 4A.

409-410. Dorsal - ventral distribution differences are detected for only 1,054 ORs. An explanation for why other ORs don't show a distribution difference should be given.

Figure panels that are too small: 3C, 3D

Reviewer #2

This manuscript describes the identification of a comprehensive list of mouse odorant receptors that respond in vivo to two different odorants, acetophenone (ACT) and trimethylthiazoline (TMT), this at a range of different concentrations. The method from which this screen results is based on a pS6-IP-Seq approach. The authors then genetically target one of the most sensitive odorant receptor candidate to ACT, Olfr923, and show that this receptor indeed responds to ACT. Finally through molecular dynamics stimulations, they identify specific Olfr923 amino acid residues that are critical for ACT binding.

This manuscript is interesting, and provides important useful about an approach (using pS6-IP-Seq) to identify the ORs that respond to a specific ligand, based on an initial publication in 2015 by the Matsunami group.

However, I have very specific and important concerns, mostly linked to methodology, in particular about their implementation of the DREAM method. I believe that the pS6-IP-Seq approach is sound, and even improves the methodology previously published. On the contrary, the way the DREAM technique is used in this manuscript is certainly not adequate. In addition, the text even misrepresents what the previously published data with DREAM showed.

Major concerns:

1) One reads in the current manuscript:“Olfr923, one of the most sensitive and robustly activated ORs against all the tested concentrations of ACT identified by pS6-IP-Seq, was not one of them (i.e. among those identified by von der Weid et al. 2015).” and again “Olfr923...was not identified as an ACT OR by the DREAM technique”. This is either falsely ingenuous, or the authors did not read the von der Weid publication with attention. Olfr923 is shown in the RNA-Seq data of Figure 3 of the von der Weid publication as a potential ACT responsive OR, in position 36 of the best responders among 1200 ORs. The authors of the von der Weid et al. study describe a list cutoff corresponding to a CI of 99% to select the best ACT receptors. Therefore, it naturally implies the presence of genuine ACT receptors at the vicinity of this cutoff. For instance, Olfr1477 is just below the cutoff (position 33) and was shown by the DREAM developers to represent a genuine ACT responsive OR. Olr923, being next to Olfr1477, is without surprise part of them. In fact, the presence of Olfr923 right next to Olfr1477 is shown in Figure 3 of von de Weid et al.

2) The authors use mice aged 20-22 days for the whole study. This is rather problematic for a DREAM approach, which is essentially based on OR expression levels. Indeed it is known that many ORs are switching in terms of levels of expression (some of them simply switch on) at the time of weaning, which is exactly 21 days. This does naturally increase variation of OR concentration between individuals and makes any modulation of OR expression due to neuronal response, problematic.

Moreover, the authors seem to ignore that DREAM, which has been shown to work in adult mice, does so poorly in immature animals. The authors of the von der Weid publication have shown that multiple times at chemosensory meetings. However, one cannot blame the authors of the present work for not having heard that. So this point is here only to make them aware of this fact.

3) The authors use the DREAM approach with three biological replicates per condition. The von der Weid publication clearly shows, by independently reevaluating by qPCR the responsive ORs identified by RNA-Seq (Figure 3), that the 3 replicates used per condition lacked statistical power. Indeed, 5 of the candidates identified by RNAseq were not confirmed by qPCR. Why then, if one wants genuinely evaluate the DREAM technique, use only three replicates for RNAseq? I would go for 5.

4) One reads in the current manuscript “The DREAM technique identifies odor responsive ORs based on a decrease in OR mRNA abundance after odor stimulation. Koerte et al found several cases where confirmed ligands did not induce a decrease in mRNA levels in the corresponding receptors in insects.” One naïve reader would think that the von der Weid publication was about insect mRNA downregulation, which it was not. In this arcticle, what was tested were almost exclusively mouse ORs (17 known OR-ligand pairs), for which false positive and false negative were evaluated to validate the approach. Both error types were found to be very low.

In the von der Weid paper, only two Drosophila OR-ligands were tested, suggesting that what was observed in vertebrate ORs could also apply to insects. However, no evaluation of the technique was provided for insect ORs, and it turned out, after the Koerte paper, that the DREAM approach is not adequate for insect ORs

5) Olfr923 has been shown in vitro, by the Matsunami group in Jiang et al. 2015, as not as sensitive to ACT as many ORs. It could even be considered, relative to these other ORs, as a poor responder. Given the expertise in OR agonist identification, of the Matsunami group, this should at least be discussed.

6) Surprisingly, the data presented for ACT responsive ORs do not seem to match with the data obtained in Jiang et al. 2015, where the best ACT responders were Olfr923, 749, 491, 136, 1377, 876, 983, 376, 1238, 1104, 57, 196, 744, 166 and 875. Among these, I can only find 3 of them in the present manuscript. But of course, since a complete list of responsive ORs is missing, one cannot really determine the overlap.

Similarly, but inversely, in the current manuscript, one of the best candidates is Olfr888 with a log2fold change that is higher than Olfr923 at 1% ACT, but was not identified by Jiang et al. 2015. Multiple additional similar examples could be added. This should be addressed, in particular since both approaches are based on the pS6-IP-Seq technique.

7) In figure 2, one clearly sees that multiple ORs identified as responsive ORs are not shared between different concentrations of the same agonist. This is of course very, very critical. To evaluate how significant this surprising lack of overlap is, and given the main aim of the present manuscript, one needs at least a comprehensive list of the responsive ORs for each agonist and at each concentration, and naturally at least a rough comparison of these lists.

8) Figure 4. I do not understand the figure. The title indicates that it corresponds to a correlation between the in vivo identified responsive ORs in vivo with the in vitro activation data, in other words an evaluation of the consistency between the two approaches. But this is not what is shown in the figure. Why look at it at a population level? To get a sense of this correlation, this analysis should be perfomed at the OR level. I suggest a graph for each agonist concentration, with on the X axis all ORs identified in vivo as responders, and on the Y axis the in vitro responses. Then, on another graph, on the X axis all ORs identified in vivo as non responders, and on the Y axis the in vitro responses. This will provide a quick sense of FDRs.

9) Figure 5. If the mapping is exclusively perfomed on ORs, as the methods suggest, then one risks a problem with normalization for the library depth, since a significant proportion of the transcripts may be downregulated after agonist exposure. Thus, if the mapping is exclusively perfomed on ORs, the approach is inadequate. Reads should be mapped on the whole transcriptome, and normalized accordingly.

10) Figure 5. Why are there raw counts shown on the Y axis? Was this not normalized? I guess they were, since otherwise the data are meaningless.

11) Figure 5-1. The authors report that using the pS6-IP-Seq approach they do not find, in their own data using the pS6-IP-Seq technique, 7 specific ORs previously found to be ACT responsive in the von der Weid paper. I guess that one could add that among these ORs not identified by the pS6-IP-Seq technique, Olfr556, Olfr922, Olfr229 and Olfr109 were shown to represent genuine ACT receptors in the von der Weid paper by colabeling the specific ORs with pS6 after ACT stimulation. One could also add that Olfr556 was previously shown, by others, to be an ACT responsive OR. Finally, one should add that among these 7 ORs, Olfr736, reported in the von der Weid paper, was identified as a genuine ACT receptor in the Matsunami paper in 2015, both by pS6-IP-Seq and by in vitro expression. I suggest, if the authors want to compare technologies, to first address the discrepancies of their results with the ones of the Matsunami group, who 4 years ago, used the very same approach, with the very same ligand.

Secondary comments:

12) Line 98, I do not understand the reference to Koerte et al relative to the “underlying differences between S6 phosphorylation and OR downregulation”

13) Line 119 “transcripts that mapped onto exons and UTRs”. UTRs are part of exons. Just non-coding parts.

14) Figure 1. Given the lower right panel, one could think that a reannotation is performed for each experiment, which is I guess, not the case.

15) Line 161. The formulation “index FASTA” is in my view inaccurate since kallisto only uses a FASTA formatted file to produce a colored de Bruijn graph that is used for pseudoalignmment wit the target transcriptome kmers. I suggest, to simplify and be relatively accurate, to write something like “index generated from a FASTA file”.

16) Why not using the whole transcriptome and map the reads exclusively on the 1100 ORs? This does not allow to buffer potential repeated elements that would be present in both ORs and other genes.

17) Figure 1F. the green color is both used for TMT and dorsal, which is confusing.

18) Figure 2 and 3. The very same volcano plots are shown. They should be removed from fig 2, or 3.

19) Line 390, I guess that what is meant is not “evolutionary conservation”, but rather “phylogenetic relationship”.

20) Line 649. Where does the number 141 come from?

## References

[B1] Altenbach C, Kusnetzow AK, Ernst OP, Hofmann KP, Hubbell WL (2008) High-resolution distance mapping in rhodopsin reveals the pattern of helix movement due to activation. Proc Natl Acad Sci USA 105:7439–7444. 10.1073/pnas.0802515105 18490656 PMC2396682

[B2] Anders S, Huber W (2010) Differential expression analysis for sequence count data. Genome Biol 11:R106. 10.1186/gb-2010-11-10-r106 20979621 PMC3218662

[B3] Anderson AK, Christoff K, Stappen I, Panitz D, Ghahremani DG, Glover G, Gabrieli JD, Sobel N (2003) Dissociated neural representations of intensity and valence in human olfaction. Nat Neurosci 6:196–202. 10.1038/nn1001 12536208

[B4] Ballesteros JA, Weinstein H (1995) Integrated methods for the construction of three-dimensional models and computational probing of structure-function relations in G protein-coupled receptors In: Methods in neurosciences, pp 366–428. San Diego: Elsevier.

[B5] Bhandawat V, Reisert J, Yau KW (2005) Elementary response of olfactory receptor neurons to odorants. Science 308:1931–1934. 10.1126/science.1109886 15976304 PMC2957801

[B6] Biever A, Valjent E, Puighermanal E (2015) Ribosomal protein S6 phosphorylation in the nervous system: From regulation to function. Front Mol Neurosci 8:75. 10.3389/fnmol.2015.00075 26733799 PMC4679984

[B7] Bozza T, Feinstein P, Zheng C, Mombaerts P (2002) Odorant receptor expression defines functional units in the mouse olfactory system. J Neurosci 22:3033–3043. 11943806 10.1523/JNEUROSCI.22-08-03033.2002 PMC6757547

[B8] Bray NL, Pimentel H, Melsted P, Pachter L (2016) Near-optimal probabilistic RNA-seq quantification. Nat Biotechnol 34:525–527. 10.1038/nbt.3519 27043002

[B9] Buck L, Axel R (1991) A novel multigene family may encode odorant receptors: A molecular basis for odor recognition. Cell 65:175–187. 10.1016/0092-8674(91)90418-x 1840504

[B10] Case DA, Darden T, Cheatham TE, Simmerling C, Roitberg A, Wang J, Duke RE, Luo R, Roe DR, Walker RC, LeGrand S, Swails J, Cerutti D, Kaus J, Betz R, Wolf RM, Merz KM, Seabra G, Janowski P, Götz AW, et al. (2014) AMBER 14. San Francisco: University of California.

[B11] Charlier L, Topin J, de March CA, Lai PC, Crasto CJ, Golebiowski J (2013) Molecular modelling of odorant/olfactory receptor complexes In: Olfactory receptors. (Crasto CJ, ed), pp 53–65. New York: Humana Press. 10.1007/978-1-62703-377-0_4 23585033

[B12] Chess A, Simon I, Cedar H, Axel R (1994) Allelic inactivation regulates olfactory receptor gene expression. Cell 78:823–834. 10.1016/s0092-8674(94)90562-2 8087849

[B13] D'Hulst C, Mina RB, Gershon Z, Jamet S, Cerullo A, Tomoiaga D, Bai L, Belluscio L, Rogers ME, Sirotin Y, Feinstein P (2016) MouSensor: A versatile genetic platform to create super sniffer mice for studying human odor coding. Cell Rep 16:1115–1125. 10.1016/j.celrep.2016.06.047 27396335

[B14] de March CA, Kim SK, Antonczak S, Goddard IIW, Golebiowski J (2015a) G protein-coupled odorant receptors: From sequence to structure. Protein Sci 24:1543–1548. 10.1002/pro.2717 26044705 PMC4570547

[B15] de March CA, Yu Y, Ni MJ, Adipietro KA, Matsunami H, Ma M, Golebiowski J (2015b) Conserved residues control activation of mammalian G protein-coupled odorant receptors. J Am Chem Soc 137:8611–8616. 10.1021/jacs.5b04659 26090619 PMC4497840

[B16] de March CA, Topin J, Bruguera E, Novikov G, Ikegami K, Matsunami H, Golebiowski J (2018) Odorant receptor 7D4 activation dynamics. Angew Chem Int Ed Engl 57:4554–4558. 10.1002/anie.201713065 29462498 PMC6268213

[B17] Dobin A, Davis CA, Schlesinger F, Drenkow J, Zaleski C, Jha S, Batut P, Chaisson M, Gingeras TR (2013) STAR: Ultrafast universal RNA-seq aligner. Bioinformatics 29:15–21. 10.1093/bioinformatics/bts635 23104886 PMC3530905

[B18] Eswar N, Webb B, Marti-Renom MA, Madhusudhan MS, Eramian D, Shen M-y, Pieper U, Sali A (2006) Comparative protein structure modeling using MODELLER In: Current protocols in bioinformatics. Hoboken, NJ: Wiley, Inc 10.1002/0471250953.bi0506s15 PMC4186674 18428767

[B19] Fried HU, Fuss SH, Korsching SI (2002) Selective imaging of presynaptic activity in the mouse olfactory bulb shows concentration and structure dependence of odor responses in identified glomeruli. Proc Natl Acad Sci USA 99:3222–3227. 10.1073/pnas.052658399 11854464 PMC122500

[B20] Godfrey PA, Malnic B, Buck LB (2004) The mouse olfactory receptor gene family. Proc Natl Acad Sci USA 101:2156–2161. 10.1073/pnas.0308051100 14769939 PMC357068

[B21] Grosmaitre X, Vassalli A, Mombaerts P, Shepherd GM, Ma M (2006) Odorant responses of olfactory sensory neurons expressing the odorant receptor MOR23: A patch clamp analysis in gene-targeted mice. Proc Natl Acad Sci USA 103:1970–1975. 10.1073/pnas.0508491103 16446455 PMC1413638

[B22] Gross-Isseroff R, Lancet D (1988) Concentration-dependent changes of perceived odor quality. Chem Senses 13:191–204. 10.1093/chemse/13.2.191

[B23] Guthrie KM, Anderson AJ, Leon M, Gall C (1993) Odor-induced increases in c-fos mRNA expression reveal an anatomical “unit” for odor processing in olfactory bulb. Proc Natl Acad Sci USA 90:3329–3333. 10.1073/pnas.90.8.3329 8475076 PMC46293

[B24] Hanchate NK, Kondoh K, Lu Z, Kuang D, Ye X, Qiu X, Pachter L, Trapnell C, Buck LB (2015) Single-cell transcriptomics reveals receptor transformations during olfactory neurogenesis. Science 350:1251–1255. 10.1126/science.aad2456 26541607 PMC5642900

[B25] Healy MD, Smith JE, Singer MS, Nadkarni PM, Skoufos E, Miller PL, Shepherd GM (1997) Olfactory receptor database (ORDB): A resource for sharing and analyzing published and unpublished data. Chem Senses 22:321–326. 10.1093/chemse/22.3.321 9218144

[B26] Ibarra-Soria X, Levitin MO, Saraiva LR, Logan DW (2014) The olfactory transcriptomes of mice. PLoS Genet 10:e1004593. 10.1371/journal.pgen.1004593 25187969 PMC4154679

[B27] Ibarra-Soria X, Nakahara TS, Lilue J, Jiang Y, Trimmer C, Souza MA, Netto PH, Ikegami K, Murphy NR, Kusma M, Kirton A, Saraiva LR, Keane TM, Matsunami H, Mainland J, Papes F, Logan DW (2017) Variation in olfactory neuron repertoires is genetically controlled and environmentally modulated. Elife 6 10.7554/eLife.21476 PMC5404925 28438259

[B28] Isogai Y, Wu Z, Love MI, Ahn MH, Bambah-Mukku D, Hua V, Farrell K, Dulac C (2018) Multisensory logic of infant-directed aggression by males. Cell 175:1827–1841.e7. 10.1016/j.cell.2018.11.032 30550786 PMC6558521

[B29] Jiang Y, Gong NN, Hu XS, Ni MJ, Pasi R, Matsunami H (2015) Molecular profiling of activated olfactory neurons identifies odorant receptors for odors in vivo. Nat Neurosci 18:1446–1454. 10.1038/nn.4104 26322927 PMC4583814

[B30] Katada S, Hirokawa T, Oka Y, Suwa M, Touhara K (2005) Structural basis for a broad but selective ligand spectrum of a mouse olfactory receptor: Mapping the odorant-binding site. J Neurosci 25:1806–1815. 10.1523/JNEUROSCI.4723-04.2005 15716417 PMC6725943

[B31] Kent WJ (2002) BLAT—the BLAST-like alignment tool. Genome Res 12:656–664. 10.1101/gr.229202 11932250 PMC187518

[B32] Kent WJ, Sugnet CW, Furey TS, Roskin KM, Pringle TH, Zahler AM, Haussler D (2002) The human genome browser at UCSC. Genome Res 12:996–1006. 10.1101/gr.229102 12045153 PMC186604

[B33] Khan AG, Parthasarathy K, Bhalla US (2010) Odor representations in the mammalian olfactory bulb. Wiley Interdiscip Rev Syst Biol Med 2:603–611. 10.1002/wsbm.85 20836051

[B34] Kida H, Fukutani Y, Mainland JD, de March CA, Vihani A, Li YR, Chi Q, Toyama A, Liu L, Kameda M, Yohda M, Matsunami H (2018) Vapor detection and discrimination with a panel of odorant receptors. Nat Commun 9:4556. 30385742 10.1038/s41467-018-06806-w PMC6212438

[B35] Knight ZA, Tan K, Birsoy K, Schmidt S, Garrison JL, Wysocki RW, Emiliano A, Ekstrand MI, Friedman JM (2012) Molecular profiling of activated neurons by phosphorylated ribosome capture. Cell 151:1126–1137. 10.1016/j.cell.2012.10.039 23178128 PMC3839252

[B36] Köster J, Rahmann S (2012) Snakemake—a scalable bioinformatics workflow engine. Bioinformatics 28:2520–2522. 10.1093/bioinformatics/bts480 22908215

[B37] Krone D, Mannel M, Pauli E, Hummel T (2001) Qualitative and quantitative olfactometric evaluation of different concentrations of ethanol peppermint oil solutions. Phytother Res 15:135–138. 10.1002/ptr.716 11268113

[B38] Langmead B, Trapnell C, Pop M, Salzberg SL (2009) Ultrafast and memory-efficient alignment of short DNA sequences to the human genome. Genome Biol 10:R25. 10.1186/gb-2009-10-3-r25 19261174 PMC2690996

[B39] Li B, Dewey CN (2011) RSEM: Accurate transcript quantification from RNA-Seq data with or without a reference genome. BMC Bioinformatics 12:323. 10.1186/1471-2105-12-323 21816040 PMC3163565

[B40] Li H, Handsaker B, Wysoker A, Fennell T, Ruan J, Homer N, Marth G, Abecasis G, Durbin R, Genome Project Data Processing S; 1000 Genome Project Data Processing Subgroup (2009) The sequence alignment/map format and SAMtools. Bioinformatics 25:2078–2079. 10.1093/bioinformatics/btp352 19505943 PMC2723002

[B41] Liberles SD, Buck LB (2006) A second class of chemosensory receptors in the olfactory epithelium. Nature 442:645–650. 10.1038/nature05066 16878137

[B42] Liu P, Jenkins NA, Copeland NG (2003) A highly efficient recombineering-based method for generating conditional knockout mutations. Genome Res 13:476–484. 10.1101/gr.749203 12618378 PMC430283

[B43] Madisen L, Zwingman TA, Sunkin SM, Oh SW, Zariwala HA, Gu H, Ng LL, Palmiter RD, Hawrylycz MJ, Jones AR, Lein ES, Zeng H (2010) A robust and high-throughput Cre reporting and characterization system for the whole mouse brain. Nat Neurosci 13:133–140. 10.1038/nn.2467 20023653 PMC2840225

[B44] Mainland JD, Lundström JN, Reisert J, Lowe G (2014) From molecule to mind: An integrative perspective on odor intensity. Trends Neurosci 37:443–454. 10.1016/j.tins.2014.05.005 24950600 PMC4119848

[B45] Malnic B, Hirono J, Sato T, Buck LB (1999) Combinatorial receptor codes for odors. Cell 96:713–723. 10.1016/s0092-8674(00)80581-4 10089886

[B46] Malnic B, Godfrey PA, Buck LB (2004) The human olfactory receptor gene family. Proc Natl Acad Sci USA 101:2584–2589. 10.1073/pnas.0307882100 14983052 PMC356993

[B47] Man O, Gilad Y, Lancet D (2004) Prediction of the odorant binding site of olfactory receptor proteins by human-mouse comparisons. Protein Sci 13:240–254. 10.1110/ps.03296404 14691239 PMC2286516

[B48] McCarthy DJ, Chen Y, Smyth GK (2012) Differential expression analysis of multifactor RNA-Seq experiments with respect to biological variation. Nucleic Acids Res 40:4288–4297. 10.1093/nar/gks042 22287627 PMC3378882

[B49] McClintock TS, Adipietro K, Titlow WB, Breheny P, Walz A, Mombaerts P, Matsunami H (2014) In vivo identification of eugenol-responsive and muscone-responsive mouse odorant receptors. J Neurosci 34:15669–15678. 10.1523/JNEUROSCI.3625-14.2014 25411495 PMC4236398

[B50] Milner ES, Do MTH (2017) A population representation of absolute light intensity in the mammalian retina. Cell 171:865–876.e6. 10.1016/j.cell.2017.09.005 28965762 PMC6647834

[B51] Miyamichi K, Serizawa S, Kimura HM, Sakano H (2005) Continuous and overlapping expression domains of odorant receptor genes in the olfactory epithelium determine the dorsal/ventral positioning of glomeruli in the olfactory bulb. J Neurosci 25:3586–3592. 10.1523/JNEUROSCI.0324-05.2005 15814789 PMC6725380

[B52] Miyamichi K, Amat F, Moussavi F, Wang C, Wickersham I, Wall NR, Taniguchi H, Tasic B, Huang ZJ, He ZG, Callaway EM, Horowitz MA, Luo LQ (2011) Cortical representations of olfactory input by trans-synaptic tracing. Nature 472:191–196. 10.1038/nature09714 21179085 PMC3073090

[B53] Mombaerts P, Wang F, Dulac C, Chao SK, Nemes A, Mendelsohn M, Edmondson J, Axel R (1996) Visualizing an olfactory sensory map. Cell 87:675–686. 10.1016/s0092-8674(00)81387-2 8929536

[B54] Moskowitz HR, Dravnieks A, Klarman LA (1976) Odor intensity and pleasantness for a diverse set of odorants. Percept Psychophys 19:122–128. 10.3758/BF03204218

[B55] Nagy A, Rossant J, Nagy R, Abramow-Newerly W, Roder JC (1993) Derivation of completely cell culture-derived mice from early-passage embryonic stem cells. Proc Natl Acad Sci USA 90:8424–8428. 10.1073/pnas.90.18.8424 8378314 PMC47369

[B56] Niimura Y, Matsui A, Touhara K (2014) Extreme expansion of the olfactory receptor gene repertoire in African elephants and evolutionary dynamics of orthologous gene groups in 13 placental mammals. Genome Res 24:1485–1496. 10.1101/gr.169532.113 25053675 PMC4158756

[B57] Oka Y, Katada S, Omura M, Suwa M, Yoshihara Y, Touhara K (2006) Odorant receptor map in the mouse olfactory bulb: In vivo sensitivity and specificity of receptor-defined glomeruli. Neuron 52:857–869. 10.1016/j.neuron.2006.10.019 17145506

[B58] O'Leary NA, Wright MW, Brister JR, Ciufo S, Haddad D, McVeigh R, Rajput B, Robbertse B, Smith-White B, Ako-Adjei D, Astashyn A, Badretdin A, Bao Y, Blinkova O, Brover V, Chetvernin V, Choi J, Cox E, Ermolaeva O, Farrell CM, et al. (2016) Reference sequence (RefSeq) database at NCBI: Current status, taxonomic expansion, and functional annotation. Nucleic acids Res 44:D733–D745. 10.1093/nar/gkv1189 26553804 PMC4702849

[B59] Parabucki A, Bizer A, Morris G, Munoz AE, Bala ADS, Smear M, Shusterman R (2019) Odor concentration change coding in the olfactory bulb. eNeuro 6 10.1523/ENEURO.0396-18.2019 PMC6397952 30834303

[B60] Ressler KJ, Sullivan SL, Buck LB (1993) A zonal organization of odorant receptor gene expression in the olfactory epithelium. Cell 73:597–609. 10.1016/0092-8674(93)90145-g 7683976

[B61] Ressler KJ, Sullivan SL, Buck LB (1994) Information coding in the olfactory system: Evidence for a stereotyped and highly organized epitope map in the olfactory bulb. Cell 79:1245–1255. 10.1016/0092-8674(94)90015-9 7528109

[B62] Robinson MD, McCarthy DJ, Smyth GK (2010) edgeR: A bioconductor package for differential expression analysis of digital gene expression data. Bioinformatics 26:139–140. 10.1093/bioinformatics/btp616 19910308 PMC2796818

[B63] Rospars JP, Lánský P, Duchamp-Viret P, Duchamp A (2000) Spiking frequency versus odorant concentration in olfactory receptor neurons. Biosystems 58:133–141. 10.1016/s0303-2647(00)00116-7 11164640

[B64] Rubin BD, Katz LC (1999) Optical imaging of odorant representations in the mammalian olfactory bulb. Neuron 23:499–511. 10.1016/s0896-6273(00)80803-x 10433262

[B65] Saito H, Chi Q, Zhuang H, Matsunami H, Mainland JD (2009) Odor coding by a mammalian receptor repertoire. Sci Signal 2:ra9. 10.1126/scisignal.2000016 19261596 PMC2774247

[B66] Saraiva LR, Ibarra-Soria X, Khan M, Omura M, Scialdone A, Mombaerts P, Marioni JC, Logan DW (2015) Hierarchical deconstruction of mouse olfactory sensory neurons: From whole mucosa to single-cell RNA-seq. Sci Rep 5:18178. 10.1038/srep18178 26670777 PMC4680959

[B67] Schneider RA, Schmidt CE, Costiloe JP (1966) Relation of odor flow rate and duration to stimulus intensity needed for perception. J Appl Physiol 21:10–14. 10.1152/jappl.1966.21.1.10 5903895

[B68] Scholz P, Kalbe B, Jansen F, Altmueller J, Becker C, Mohrhardt J, Schreiner B, Gisselmann G, Hatt H, Osterloh S (2016) Transcriptome analysis of murine olfactory sensory neurons during development using single cell RNA-Seq. Chem Senses 41:313–323. 10.1093/chemse/bjw003 26839357

[B69] Schrödinger L (2013) 1: Maestro, version 9.4. New York: Schrödinger, LLC.

[B70] Sirotin YB, Shusterman R, Rinberg D (2015) Neural coding of perceived odor intensity. eNeuro 2 10.1523/ENEURO.0083-15.2015 PMC4672005 26665162

[B71] Sosulski DL, Bloom ML, Cutforth T, Axel R, Datta SR (2011) Distinct representations of olfactory information in different cortical centres. Nature 472:213–216. 10.1038/nature09868 21451525 PMC3354569

[B72] Stettler DD, Axel R (2009) Representations of odor in the piriform cortex. Neuron 63:854–864. 10.1016/j.neuron.2009.09.005 19778513

[B73] Storace DA, Cohen LB (2017) Measuring the olfactory bulb input-output transformation reveals a contribution to the perception of odorant concentration invariance. Nat Commun 8:81. 10.1038/s41467-017-00036-2 28724907 PMC5517565

[B74] Tan L, Xie XS (2018) A near-complete spatial map of olfactory receptors in the mouse main olfactory epithelium. Chem Senses 43:427–432. 10.1093/chemse/bjy030 29796642 PMC6454507

[B75] Tan L, Li Q, Xie XS (2015) Olfactory sensory neurons transiently express multiple olfactory receptors during development. Mol Syst Biol 11:844. 10.15252/msb.20156639 26646940 PMC4704490

[B76] Topin J, de March CA, Charlier L, Ronin C, Antonczak S, Golebiowski J (2014) Discrimination between olfactory receptor agonists and non-agonists. Chemistry 20:10227–10230. 10.1002/chem.201402486 25043138

[B77] Trott O, Olson AJ (2010) AutoDock Vina: Improving the speed and accuracy of docking with a new scoring function, efficient optimization, and multithreading. J Comput Chem 31:455–461. 10.1002/jcc.21334 19499576 PMC3041641

[B78] Uchida N, Mainen ZF (2007) Odor concentration invariance by chemical ratio coding. Front Syst Neurosci 1:3. 10.3389/neuro.06.003.2007 18958244 PMC2526272

[B79] Vassar R, Ngai J, Axel R (1993) Spatial segregation of odorant receptor expression in the mammalian olfactory epithelium. Cell 74:309–318. 10.1016/0092-8674(93)90422-m 8343958

[B80] Vassar R, Chao SK, Sitcheran R, Nuñez JM, Vosshall LB, Axel R (1994) Topographic organization of sensory projections to the olfactory bulb. Cell 79:981–991. 10.1016/0092-8674(94)90029-9 8001145

[B81] von der Weid B, Rossier D, Lindup M, Tuberosa J, Widmer A, Col JD, Kan C, Carleton A, Rodriguez I (2015) Large-scale transcriptional profiling of chemosensory neurons identifies receptor-ligand pairs in vivo. Nat Neurosci 18:1455–1463. 10.1038/nn.4100 26322926

[B82] Wachowiak M, Cohen LB (2001) Representation of odorants by receptor neuron input to the mouse olfactory bulb. Neuron 32:723–735. 10.1016/s0896-6273(01)00506-2 11719211

[B83] Warming S, Costantino N, Court DL, Jenkins NA, Copeland NG (2005) Simple and highly efficient BAC recombineering using galK selection. Nucleic Acids Res 33:e36. 10.1093/nar/gni035 15731329 PMC549575

[B84] Wilson CD, Serrano GO, Koulakov AA, Rinberg D (2017) A primacy code for odor identity. Nat Commun 8:1477. 10.1038/s41467-017-01432-4 29133907 PMC5684307

[B85] Wingler LM, McMahon C, Staus DP, Lefkowitz RJ, Kruse AC (2019) Distinctive activation mechanism for angiotensin receptor revealed by a synthetic nanobody. Cell 176:479–490.e2. 10.1016/j.cell.2018.12.006 30639100 PMC6367718

[B86] Yu YQ, de March CA, Ni MJJ, Adipietro KA, Golebiowski J, Matsunami H, Ma MH (2015) Responsiveness of G protein-coupled odorant receptors is partially attributed to the activation mechanism. Proc Natl Acad Sci USA 112:14966–14971. 10.1073/pnas.1517510112 26627247 PMC4672800

[B87] Zhang XM, Firestein S (2002) The olfactory receptor gene superfamily of the mouse. Nat Neurosci 5:124–133. 10.1038/nn800 11802173

